# Role of informal sector to combat unemployment in developing economy: A modeling study

**DOI:** 10.1016/j.heliyon.2024.e33378

**Published:** 2024-06-27

**Authors:** A.K. Misra, Mamta Kumari, Mohammad Sajid

**Affiliations:** a Department of Mathematics, Institute of Science, Banaras Hindu University, Varanasi – 221 005, India; b Department of Mechanical Engineering, College of Engineering, Qassim University, Saudi Arabia

**Keywords:** Unemployment, Informal sector, Mathematical model, Sensitivity analysis, Simulation, Bifurcation, Optimal control

## Abstract

In developing countries, informal sector is the primary job provider for a significant portion of the workforce. This study aims to analyze how jobs in the informal sector affect the unemployment dynamics of developing nations. To achieve this goal, we formulate a nonlinear mathematical model by categorizing the considered workforce into three distinct classes: unemployed, employed, and self-employed, and include a separate dynamic variable to represent vacancies within the informal sector. The proposed model is analyzed using the qualitative theory of dynamical systems. A threshold quantity known as the reproduction number is derived and using this, one can compute the job generation rate necessary to stabilize the system. It is observed that variations in the reproduction number lead to qualitative changes, such as transcritical (forward or backward) and saddle-node bifurcations in the formulated system. Moreover, we propose an optimal control problem to determine an optimal strategy for government policy implementation in enhancing the employment rate of unemployed individuals and promoting the self-employment of informal employees inside the informal sector. Further, the analytical findings are validated numerically. The obtained results suggest that promoting the self-employment of informal employees for job generation effectively reduces unemployment.

## 1 Introduction

**Figure 1:**
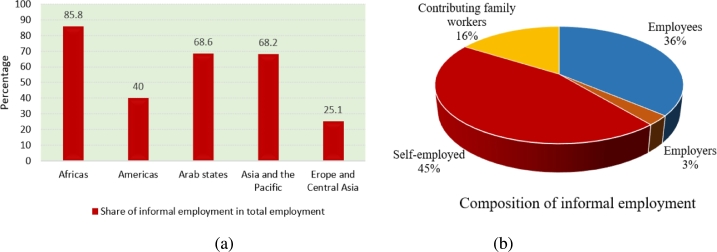
(a) Share of different regions in informal employment (b) Composition of informal employment [3].

Unemployment is a major concern in developing countries. The growing population and limited formal job opportunities are amplifying the percentage of unemployed individuals in total workforce. In these situations, jobs in the informal sector become the primary source of income for unemployed individuals to meet their basic needs, such as food, shelter, clothing, household expenses, etc. Today, more than 60% of the world's employed workforce earn their livelihood through the informal economy [1]. This sector encompasses a diverse group of laborers, unregistered self-regulated businesses, and small enterprises employing fewer than ten employees [2]. The statistical report of ILO (International Labor Organization) reveals that this form of employment is widespread across all regions of the world, regardless of their socio-economic status (see Fig. 1(a)). However, its prevalence is notably higher in developing nations compared to developed ones [3]. The inflexibility of the formal job market is one of the main reasons for the proliferation of this sector. Apart from this, the inoperable enforcement systems regarding registration and taxation and inadequate regulatory frameworks of developing nations also foster the growth of informal sector [4]. According to the ILO employment report, a large share of new jobs in developing countries is generated by self-operated informal businesses that employ millions of workers [1]. These businesses are not subjected to the same regulations as formal businesses, which makes them more flexible for workers. Its flexible nature provides opportunities for employment, income generation, and entrepreneurship, particularly for marginalized groups [5]. The reliance of a significant portion of the workforce on informal employment becomes incredibly apparent during periods of economic uncertainty, such as the COVID-19 pandemic. It has been observed that in India, 78% of informal workers had lost their livelihoods during the COVID-19 lockdown, and approximately 82% of informal workers were faced with food insecurity [6]. Many informal businesses were closed during this period, leading to significant economic losses for the government.

Some empirical studies have shown that informal jobs provide a base for less-educated individuals and facilitate career reform because the work experiences gained through informal jobs enhance their employability [7], [8], [9], [10]. While working in the informal sector, individuals face several difficulties, such as long working hours, low wages, job insecurity, and limited opportunities for promotion. On the other hand, they also observe the profits made by business owners, which motivates them to start their own businesses and become self-employed [11], [12], [13], [14]. At the global level, self-employed individuals constitute 45% of informal employment, while the share of wage employees is 36% (Fig. 1(b)). The remaining 19% of informal employment is accounted to family workers and employers [3]. It is evident that a significant portion of informal employment consists of self-employed individuals; therefore, this sector is also referred to as the sector of self-employed individuals. Apart from this, there are evidences in the literature confirming that a bidirectional causality exists between informal employment and economic growth in developing countries [15], [16]. By absorbing surplus labor, fostering innovation, and meeting unmet demand, the informal sector can contribute to overall economic expansion. Therefore, the governments of several developing countries are directing their attention towards strengthening the productivity of this sector. For instance, the government of India has recently initiated several schemes such as PM SVANidhi (The Prime Minister Street Vendor's AtmaNirbhar Nidhi) scheme [17], PM-SYM (Pradhan Mantri Shram Yogi Maan-dhan) [18], etc., to strengthen the informal sector for generating more jobs. The informal sector's contribution in employing a large proportion of the workforce and encouraging self-employment makes it crucial from a development perspective. However, the heterogeneity of this sector poses a challenge to policymakers in devising effective and targeted policies. In this context, mathematical modeling may help in identifying crucial factors associated with this sector, which can drive unemployment reduction in developing nations.

### 1.1 Literature review of some existing mathematical models for unemployment

Mathematical modeling provides valuable insights necessary to tackle social challenges without making extensive surveys and field experimentation. In the past, many researchers have formulated mathematical models for analyzing the problem of unemployment by considering different factors, such as vacancy generation, skill development, impact of highly skilled individuals, industrialization, financial crises, policy implementation, etc. In this context, Misra and Singh [19] formulated a mathematical model by assuming unemployed persons, temporarily employed persons, and regularly employed persons as dynamic variables. Their study demonstrates that how the transition from temporary to regular employment can either increase or decrease unemployment, depending on specific model parameters. Furthermore, the authors of [20] presented a mathematical model to reduce unemployment by generating vacancies proportional to the number of unemployed individuals. This study concluded that while generating vacancies can reduce unemployment, delaying this process may destabilize the system. Additionally, some optimal control problems have been formulated to analyze how government policies for providing employment and generating vacancies can be efficiently implemented to reduce unemployment levels [21], [22]. In these studies, some optimal strategies are discussed that benefit policymakers in combating unemployment. Furthermore, Harding and Nimtu [23] emphasized in framing employment policies using a mathematical model in the presence of migrant workers. Their findings indicate that alleviating unemployment requires creating job opportunities in proportion to both the local unemployed population and migrant workers of considered region. In this continuation, some researchers have investigated the effect of skill development on reducing unemployment using mathematical models [24], [25]. In [24], the authors have clarified that as the effectiveness of skill development improves, the number of unemployed individuals decreases in the considered region. Additionally, the authors of [25] examined the impact of skill development and the highly skilled individuals' contribution to unemployment alleviation. They found that highly skilled individuals play a pertinent role in reducing the unemployment burden of society. Furthermore, the authors of [26] have proposed a delayed mathematical model in which job vacancies are assumed to be generated either in proportion to the number of unemployed individuals or as a result of retirements or terminations of regular employees. They have found that for any value of delay parameter, the coexisting equilibrium remains nonlinearly stable under certain conditions. Moreover, a mathematical model incorporating distributed delay in the generation of vacancies was introduced in [27] to investigate unemployment dynamics. Their findings indicate that the coexisting equilibrium is globally stable and independent of the size of initial perturbation or considered delay kernel. Furthermore, the authors of [28] have formulated a mathematical model to address the unemployment problem in developing countries, where job opportunities are limited, and have examined the impact of training programs. Their results reveal that training programs play a beneficial role in enhancing the skills of unemployed population, which reduces unemployment. Furthermore, Singh et al. [29] presented a mathematical model to analyze the impact of unemployment-augmented industrialization on unemployment and obtained that if the government increases the growth of industries in proportion to the unemployed population, then it is much more effective to reduce unemployment. Further, Ashi et al. [30] also clarified through their modeling analysis that the generation of vacancies in proportion to unemployed individuals is advantageous in controlling unemployment. Moreover, in [31], the interaction between structural and cyclical unemployment has been studied using a mathematical model. Their findings assist public authorities in simulating the impact of various economic policies, which can help unemployed individuals to recover from cyclical unemployment and prevent them from falling into structural unemployment. Further, some researchers have conducted modeling studies to explore the impact of financial crises on unemployment [32], [33]. In the article [32], the authors observed that a high self-employment rate can mitigate unemployment even during financial crises. On the other hand, the study presented in [33] provides theoretical confirmation of Okun's law. Building upon the work of [20], Rajpal et al. [34] conducted further analysis to examine the combined impact of delays in vacancy generation and skill development on unemployment. They have also discussed the effects of early retirement on employed individuals. Their findings highlight that both the factors, vacancy generation and skill development, effectively reduce unemployment. Recently, the authors of [35] have presented a mathematical model to analyze the impact of informal skill learning in getting the regular employment. In this article, authors have considered that individuals of the unemployed class either directly move to the regular employed class or work for some time in the informal sector to acquire skills and then join the regular employed class. Further, they considered that after acquiring skills, a fraction of individuals of informal sector contribute to generate regular employment. Their results unveil that informal skill learning provided by small businesses in the informal sector is effective to lowering the unemployment level of unskilled individuals.

From the above-discussed literature, it can be noted that most of the existing mathematical models have primarily focused on addressing unemployment by generating job opportunities [20], [21], [22], [23], [26], [30] and offering formal skill training that aligns with industry needs [24], [25], [28], [34]. However, there has been limited exploration of the informal sector's role, which employs a significant portion of the workforce and offers learning opportunities to less educated or unskilled individuals [35]. This underscores a significant gap in mathematical modeling concerning the impact of informal sector employment on reducing unemployment. Thus, there is a notable gap in the field of mathematical modeling regarding the contribution of informal sector jobs to unemployment reduction. This gap emphasizes the importance of our research in addressing this critical void. The present study focuses solely on the informal sector, aiming to evaluate the impact of informal sector employment on reducing unemployment in developing nations. The rest of the paper is structured as follows: In section 2, we present the formulation of a mathematical model. Section 3 discusses the feasibility of equilibria and provides a comprehensive definition of reproduction number for the proposed system. Sections 4 and 5 cover the local properties of the obtained equilibria in terms of their local stability and bifurcation phenomena, respectively. Further, section 6 delves into analyzing the global stability of interior equilibrium. In section 7, we introduce an optimal control problem for our proposed model system and discuss its findings. Section 8 presents the numerical validation of the obtained results, and section 9 concludes the paper. Finally, section 10 provides an overview of our work's advantages, limitations, and future directions.

## 2 Mathematical model

In our model formulation, we have divided the informal sector workforce into three classes. The first is the unemployed class (U(t)), representing individuals susceptible to informal employment. The second is the employed class (E(t)), which represents informal employees who work at businesses regulated by self-employed individuals. The third is the self-employed class (S(t)), representing individuals who have their own businesses and work independently. To represent the vacancies available in the informal sector, we have considered a separate dynamical variable V(t). Here, we have considered only those vacancies that are created by self-regulated businesses without any financial support of the government or formal private sector. Further, we proceed with the following assumptions regarding the rate of changes for the considered dynamical variables:

(1) Individuals above the minimum age to join the workforce and actively searching for employment enter the unemployed class *U* at a constant rate *A*. Further, individuals exit from each considered class at rate *d* due to cumulative effects of natural death and emigration.
(2) The movement of individuals from class *U* to *E* depends on the available vacancies in the informal sector and the number unemployed individuals. Therefore, we consider that unemployed individuals move from class *U* to *E* at a rate *kUV*, where *k* is the proportionality constant known as the employment rate coefficient of unemployed individuals.
(3) We consider that after getting work experiences from their informal jobs and motivation from their business owner or self-employed community, some informal employees move towards self-employment. Thus, informal employees move from class *E* to *S* in two ways: (i) they shift willingly towards self-employment at a rate *rE* and join the class *S*, and (ii) they are motivated/inspired by the thriving community of self-employed individuals and move towards self-employment at a rate *γSE* and join the class *S*.
(4) When informal businesses face financial losses, some informal employees are terminated from their jobs at a rate *θE* and some self-employed individuals close their entire businesses at a rate *νS* and revert to unemployed class *U*. Apart from this, some informal employees choose to leave their jobs voluntarily at a rate *αE* to seek alternative employment opportunities outside the informal sector.
(5) Vacancies within the informal sector increase in two ways: (i) when self-employed individuals create job opportunities for other job seekers at a rate *ϕS* based on the needs of their self-regulated businesses, and (ii) when informal employees leave their positions due to resignation and natural death, occurring at a rate (α+d)E. Additionally, these vacancies decrease at a rate *δV* due to financial constraints faced by self-employed individuals and at a rate of *kUV* when unemployed individuals fill them [32].

**Figure 2:**
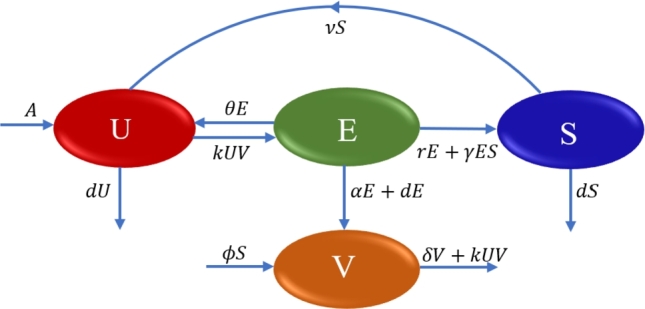
Schematic diagram for model system (1).

Based on all the above assumptions, a schematic diagram for the developed model system is represented in Fig. 2 and corresponding set of differential equations is given as follows:

(1) $$\left\{ \begin{array}{cc} \frac{dU}{dt} & =A-kUV+\theta E+\nu S-dU,\\ \frac{dE}{dt} & =kUV-(\theta +\alpha +d)E-rE-\gamma SE,\\ \frac{dS}{dt} & =rE+\gamma SE-\nu S-dS,\\ \frac{dV}{dt} & =\phi S+(\alpha +d)E-\delta V-kUV, \end{array}\right.$$

with initial conditions $U(0)=U_{0}>0$, $E(0)=E_{0}\geq 0$, $S(0)=S_{0}\geq 0$ and $V(0)=V_{0}\geq 0$. The following set contains the region of attraction for the system (1), in which system remains positive and bounded for all time t>0.

(2) $$\Omega =\left\{(U,E,S,V)\in R_{+}^{4}:0<U+E+S\leq \frac{A}{d},0<V\leq \frac{(\phi +\alpha +d)A}{\delta d}\right\}.$$

## 3 Equilibrium analysis

Equilibrium represents a solution of the system that remains unchanged on varying time and can be obtained by solving the subsequent set of algebraic equations:

(3) A−kUV+θE+νS−dU=0,

(4) kUV−(θ+α+d)E−rE−γSE=0,

(5) rE+γSE−νS−dS=0,

(6) ϕS+(α+d)E−δV−kUV=0.

From the equation (5), we have

(7) $$S=\frac{rE}{\nu +d-\gamma E}.$$

Here, it can be noted that S>0 when E<(ν+d)/γ. Now, adding equations (3) and (4), we get

(8) A−(α+d)E+νS−rE−γSE−dU=0.

Using the expression of *S* from equation (7) in equation (8), we get *U* in terms of *E* as

(9) $$U=\frac{A}{d}-\left(\frac{\alpha +d}{d}\right)E-\frac{rE}{\nu +d-\gamma E}=H(E)\;(say).$$

The observable facts for H(E) are: (*i*) H(0)=A/d>0, (ii) $\lim_{E\to \frac{\nu +d}{\gamma }+}H(E)=-\infty$, and (iii) $H^{\prime }(E)<0$. Thus, H(E)=0 has a unique positive root (say $\overset{ˆ}{E}$) in the interval (0,(ν+d)/γ) and H(E)>0 when $E\in (0,\overset{ˆ}{E})$. Hence for the positive value of *U*, the value of *E* must lie in the interval $\left(0,\overset{ˆ}{E}\right)$. Now, from the equation (6), we have

(10) $$V=\frac{\phi S+(\alpha +d)E}{\delta +kU}=\frac{\left(\frac{\phi r}{\nu +d-\gamma E}+(\alpha +d)\right)E}{\delta +k\left(\frac{A}{d}-\frac{(\alpha +d)E}{d}-\frac{rE}{\nu +d-\gamma E}\right)}.$$

Now, using the values of *S*, *U* and *V* from equations (7), (9) and (10), respectively in equation (4), we get the following equation in *E*

(11) $$\overset{˜}{F}(E)=\frac{k\left(\frac{A}{d}-\frac{(\alpha +d)E}{d}-\frac{rE}{\nu +d-\gamma E}\right)\left(\frac{\phi r}{\nu +d-\gamma E}+(\alpha +d)\right)E}{\delta +k\left(\frac{A}{d}-\frac{(\alpha +d)E}{d}-\frac{rE}{\nu +d-\gamma E}\right)}-(\theta +\alpha +d+r)E-\frac{\gamma rE^{2}}{\nu +d-\gamma E}=0.$$

From equation (11), we have two possible cases:

### 3.1 The vacancy-free equilibrium and reproduction number

If E=0, we obtain a unique boundary equilibrium $W_{0}(A/d,0,0,0)$, say *vacancy-free equilibrium* (VFE), which denotes that state when vacancies are not generated in the informal sector and the entire considered workforce is unemployed. Using next-generation matrix method [36], we define a threshold quantity at equilibrium $W_{0}(A/d,0,0,0)$:

(12) $$R_{0}=k\frac{A/d}{\left(\theta +\alpha +d+r\right)}\frac{\left(\frac{\phi r}{\nu +d}+(\alpha +d)\right)}{\left(\delta +kA/d\right)},$$

which is known as the *reproduction number* for the model system (1). Firstly, complete comprehension is necessary to derive the physical interpretation of threshold $R_{0}$. The quantity r/(ν+d) represents the average number of informal employees who switch to self-employment, where *r* is the movement rate of individuals from informal employment to self-employment, and 1/(ν+d) is the average time spent by an individual in the self-employed class. Further, a self-employed individual generates jobs in the informal sector at a rate ϕr/(ν+d). Additionally, vacancies arise from the resignation and natural death of employed individual at a rate (α+d). Hence, a total ϕr/(ν+d)+(α+d) number of vacancies are created in unit time within the informal sector. The term 1/(δ+kA/d) corresponds to the average period during which these created vacancies remain vacant. Therefore, the ratio (ϕr/(ν+d)+(α+d))/(δ+kA/d) represents the average cumulated number of vacancies created in the informal sector. As *k* is the employment rate of unemployed individuals and A/d is the total number of unemployed individuals who are susceptible to fill these vacancies, the expression (kA/d)((ϕr/(ν+d)+α+d)/(δ+kA/d)) denotes the number of unemployed individuals who secure employment in unit time. Since 1/(θ+α+d+r) is the average time spent by an employed individual in the employed class *E*, therefore, the ratio $R_{0}$ represents the average number of individuals getting employment due to the average number of vacancies created within the informal sector, in a total susceptible unemployed population.

### 3.2 Interior equilibria

If E≠0, the interior equilibrium can be obtained by finding positive roots of the following equation,

(13) $$F(E)=\frac{k\left(\frac{A}{d}-\frac{(\alpha +d)E}{d}-\frac{rE}{\nu +d-\gamma E}\right)\left(\frac{\phi r}{\nu +d-\gamma E}+(\alpha +d)\right)}{\delta +k\left(\frac{A}{d}-\frac{(\alpha +d)E}{d}-\frac{rE}{\nu +d-\gamma E}\right)}-(\theta +\alpha +d+r)-\frac{\gamma rE}{\nu +d-\gamma E}=0.$$

We observe that function F(E) has at most three real roots. Furthermore, we have the following observations for F(E):

*(i)* $\lim_{E\to -\infty }F(E)=-\infty \;\;\text{and}\;\;\lim_{E\to +\infty }F(E)=+\infty$.
*(ii)* $F(0)=(\theta +\alpha +d+r)(R_{0}-1)$.
*(iii)* $F(\overset{ˆ}{E})=-(\theta +\alpha +d+r)-\frac{r\gamma \overset{ˆ}{E}}{\left(\nu +d-\gamma \overset{ˆ}{E}\right)}<0$.

**Figure 3:**
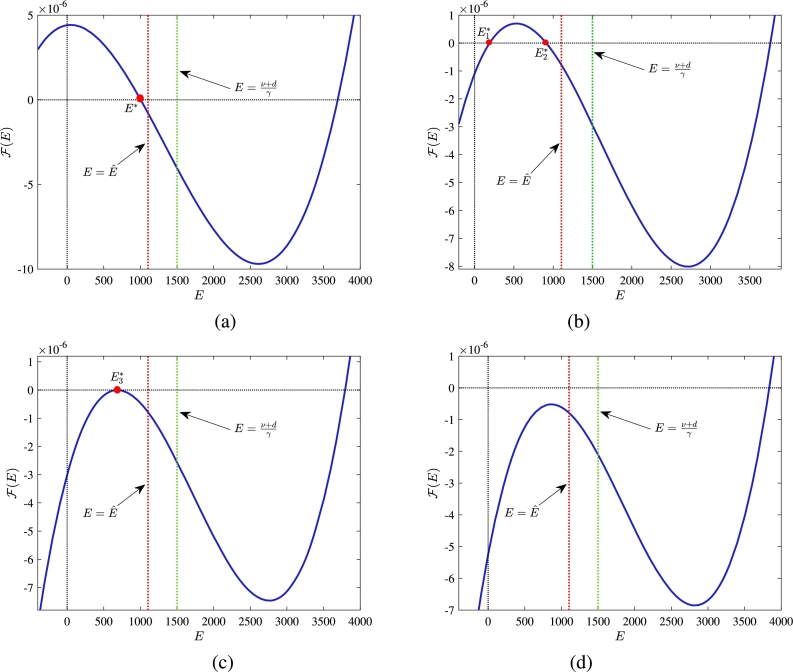
Graph of F(E) for (a) $R_{0}>1$ and (b)-(d) $R_{0}<1$.
(2) If $R_{0}<1$ then F(0)<1, thus we get three possible scenarios for the positive roots of F(E) in the interval $\left(0,\overset{ˆ}{E}\right)$: (i) has two positive roots, say $E_{1}^{⁎}$ and $E_{2}^{⁎}$ (Fig. 3(*b*)), (ii) has one positive root, say $E_{3}^{⁎}$ (Fig. 3(*c*)) and (iii) has no positive root (Fig. 3(*d*)).

Thus, we have two possible cases regarding the feasibility of positive roots of F(E)=0 in the interval $\left(0,\overset{ˆ}{E}\right)$:

(1) If $R_{0}>1$ then F(0)>0, thus one of the roots of F(E) is consistently negative. Among the remaining two real roots, one lies within the interval $\left(0,\overset{ˆ}{E}\right)$ (Fig. 3(*a*)), and the other always lies outside this interval. Therefore, for $R_{0}>1$, the equation F(E)=0 has a unique positive real solution (say $E^{⁎}$) within the specified interval $\left(0,\overset{ˆ}{E}\right)$.

Using the obtained positive value of *E*, we can get the other components of concerning equilibrium. Therefore, we conclude the outcomes related to the feasibility of interior equilibrium through the theorem presented below.

Theorem 1 *The formulated system*(1)*has*

(*i*) *unique interior equilibrium if*$R_{0}>1$*, and*
(ii) *two or one or no interior equilibrium if*$R_{0}<1$*.*

Remark 1 When $R_{0}>1$, then we observe that $dE^{⁎}/dk>0$, $dS^{⁎}/dk>0$, $dV^{⁎}/dk>0$ and $dU^{⁎}/dk<0$. It indicates that if on average more than one unemployed individuals are getting employment through the average number of vacancies generated by a self-employed individual and positions vacated by employed individuals in informal sector, then by increasing the employment rate coefficient of unemployed individuals, we can effectively reduce the equilibrium level of number of unemployed individuals in the community.

## 4 Stability analysis

To investigate the local stability behavior of the equilibrium, we linearize the system in the neighborhood of concerning equilibrium. Further, we analyze the sign of the real parts of the eigenvalues belonging to the associated Jacobian matrix. If these real parts are negative, we can conclude that the equilibrium is locally asymptotically stable.

**Stability of** $W_{0}(A/d,0,0,0)$ **:**

The Jacobian matrix for the system (1) is obtained as

$$J=\begin{array}{c} \left[\begin{array}{cccc} -(kV+d)\; & \theta \; & \nu \; & -kU\\ kV\; & -(\theta +\alpha +d+r+\gamma S)\; & -\gamma E\; & kU\\ 0\; & r+\gamma S\; & -(\nu +d-\gamma E)\; & 0\\ -kV\; & \alpha +d\; & \phi \; & -(\delta +kU) \end{array}\right]. \end{array}$$

The Jacobian matrix at equilibrium $W_{0}(A/d,0,0,0)$ is obtained as

$$J_{W_{0}}=\begin{array}{c} \left[\begin{array}{cccc} -d\; & \theta \; & \nu \; & -kA/d\\ 0\; & -(\theta +\alpha +d+r)\; & 0\; & kA/d\\ 0\; & r\; & -(\nu +d)\; & 0\\ 0\; & \alpha +d\; & \phi \; & -(\delta +kA/d) \end{array}\right]. \end{array}$$

The characteristic equation of the above matrix is given by the following equation

(14) $$(d+\varphi )(\varphi^{3}+B_{1}\varphi^{2}+B_{2}\varphi +B_{3})=0,$$

where $B_{1}=\theta +\alpha +r+\nu +2d+\delta +kA/d$, $B_{2}=(\theta +\alpha +d+r)(\nu +d+\delta )+(\theta +r)kA/d+(\nu +d)kA/d$ and $B_{3}=(\nu +d)(\theta +\alpha +d+r)(\delta +kA/d)(1-R_{0})$.

We observe that one eigenvalue is −*d* and remaining three are the roots of cubic polynomial $\varphi^{3}+B_{1}\varphi^{2}+B_{2}\varphi +B_{3}=0$. It is evident that $B_{1}$ and $B_{2}$ are positive. Moreover, $B_{3}$ is positive when $R_{0}<1$, and conversely negative when $R_{0}>1$. After some simplifications, we obtain that $B_{1}B_{2}-B_{3}>0$. Thus, by the *Routh-Hurwitz criterion*, it can be concluded that equation (14) has negative roots or roots containing negative real part if $R_{0}<1$. Therefore, we provide the result regarding stability of $W_{0}$ through the following theorem.

Theorem 2 *The equilibrium*$W_{0}$*is locally asymptotically stable if*$R_{0}<1$*and unstable if*$R_{0}>1$*.*

**Stability of** $W^{⁎}(U^{⁎},E^{⁎},S^{⁎},V^{⁎})$ **:**

The Jacobian matrix at interior equilibrium $W^{⁎}$ is

$$J{|}_{W^{⁎}}=\begin{array}{c} \left[\begin{array}{cccc} -c_{11}\; & \theta \; & \nu \; & -kU^{⁎}\\ kV^{⁎}\; & -c_{22}\; & -\gamma E^{⁎}\; & kU^{⁎}\\ 0\; & c_{32}\; & -c_{33}\; & 0\\ -kV^{⁎}\; & c_{42}\; & \phi \; & -c_{44} \end{array}\right], \end{array}$$

where $c_{11}=kV^{⁎}+d$, $c_{22}=\theta +\alpha +d+r+\gamma S^{⁎}$, $c_{32}=r+\gamma S^{⁎}$, $c_{33}=\nu +d-\gamma E^{⁎}$, $c_{42}=\alpha +d$, and $c_{44}=\delta +kU^{⁎}$. The characteristic polynomial of the above matrix is obtained as

(15) $$\psi^{4}+C_{1}\psi^{3}+C_{2}\psi^{2}+C_{3}\psi +C_{4}=0,$$

where

$$\begin{array}{cc} C_{1}= & c_{11}+c_{22}+c_{33}+c_{44},\\ C_{2}= & c_{11}c_{22}+c_{11}c_{33}+c_{11}c_{44}+c_{22}c_{33}+c_{22}c_{44}+c_{33}c_{44}-k^{2}U^{⁎}V^{⁎}-\theta kV^{⁎}+\gamma E^{⁎}c_{32}-kU^{⁎}c_{42},\\ C_{3}= & c_{11}c_{22}c_{33}+c_{11}c_{22}c_{44}+c_{11}c_{33}c_{44}+c_{22}c_{33}c_{44}+k^{2}U^{⁎}V^{⁎}(\theta +c_{42})+\gamma E^{⁎}c_{32}(c_{11}+c_{44})-kU^{⁎}(\phi c_{32}+c_{42}c_{11}+c_{42}c_{33})\\ & -\theta kV^{⁎}(c_{33}+c_{44})-k^{2}U^{⁎}V^{⁎}(c_{22}+c_{33})-\nu kV^{⁎}c_{32},\\ C_{4}= & c_{11}c_{22}c_{33}c_{44}+\gamma E^{⁎}c_{11}c_{32}c_{44}+k^{2}U^{⁎}V^{⁎}(\phi c_{32}++\nu c_{32}+\theta c_{33}+c_{42}c_{33})-k^{2}U^{⁎}V^{⁎}(c_{22}c_{33}+\gamma E^{⁎}c_{32})\\ & -kV^{⁎}c_{44}(\theta c_{33}+\nu c_{32})-kU^{⁎}c_{11}(\phi c_{32}+c_{42}c_{33}). \end{array}$$

It is clear that $C_{1}>0$ and $C_{2}>0$. After some algebraic simplifications, we get that $C_{3}$ and $C_{4}$ both are positive if $(\delta +kU^{⁎})(\nu +d)>\phi kU^{⁎}$. For $R_{0}>1$, the inequality $(\delta +kU^{⁎})(\nu +d)>\phi kU^{⁎}$ is always satisfied. Thus, by using *Routh-Hurwitz criterion*, it can be concluded that equation (15) has either negative roots or roots having negative real parts if $C_{3}(C_{1}C_{2}-C_{3})-C_{1}^{2}C_{4}>0$. Thus, the result concerning the local stability of $W^{⁎}$ is concluded in the following theorem.

Theorem 3 *When*$R_{0}>1$*, the interior equilibrium*$W^{⁎}$*is locally asymptotically stable under the condition*$C_{3}(C_{1}C_{2}-C_{3})-C_{1}^{2}C_{4}>0$*.*

## 5 Bifurcation analysis

### 5.1 Transcritical bifurcation

From Theorem 2, we observe that equilibrium $W_{0}$ changes its stability at $R_{0}=1$. Thus, the existence of transcritical bifurcation is possible at $R_{0}=1$. The following theorem summarizes our conclusion regarding the occurrence of transcritical bifurcation.

Theorem 4 *The model system*(1)*undergoes transcritical bifurcation at*$R_{0}=1$*around the equilibrium*$W_{0}$*in forward direction if*$\gamma <\gamma^{⁎}$*and in the backward direction if*$\gamma >\gamma^{⁎}$*.*

The proof of above Theorem 4 and expression of $\gamma^{⁎}$ are provided in **Appendix** A.

### 5.2 Saddle-node bifurcation

In subsection 3.2, we have obtained that if $R_{0}<1$, three scenarios are possible regarding the feasibility of interior equilibrium. The system (1) may have two, one or none interior equilibrium according to the variation in the value of $R_{0}$ between zero and one. Let $0<R_{0}^{⁎}<1$ be that critical value at which system (1) has unique interior equilibrium ($W_{3}^{⁎}$). Using the Sotomayor's Theorem given in [37], we affirm the theorem stated below for ensuring the emergence of saddle-node bifurcation.

Theorem 5 *The developed system*(1)*exhibits saddle-node bifurcation at*$R_{0}=R_{0}^{⁎}$*if*$S_{2}\neq 0$*.*

The proof of above Theorem 5 and description of the expression $S_{2}$ are provided in **Appendix** B.

## 6 Global stability

Using the Lyapunov's stability theory, the resulting conclusion regarding the global stability of $W^{⁎}$ is presented below.

Theorem 6 *Equilibrium*$W^{⁎}$*is globally asymptotically stable in the region* Ω *(defined in* (2)*) if the following inequalities hold*

(16) $$\frac{kU^{⁎}}{\delta }(\theta +\alpha +d+r+\gamma S^{⁎})E^{⁎}\leq dU^{⁎},$$

(17) $$\left(\nu +\phi \frac{kU^{⁎}}{\delta }\right)S^{⁎}\leq \left(1+\frac{kU^{⁎}}{\delta }\right)rE^{⁎}.$$

The proof of above mentioned Theorem 6 is conferred in **Appendix** C.

• Inequality (16) can be written as
  $$\begin{array}{c} \left(\frac{kE^{⁎}}{\delta }\right)\left(\frac{1}{d}\right)\leq \frac{1}{\theta +\alpha +d+r+\gamma S^{⁎}}, \end{array}$$
  which shows that for the stability of interior equilibrium, the weighted average time spent by individuals in the employed class is greater than or equal to the weighted average life expectancy.
• Similarly, inequality (17) can be written as
  $$\begin{array}{c} \frac{\delta }{\delta +kU^{⁎}}\leq \frac{r}{\left(\nu +\phi \frac{kU^{⁎}}{\delta }\right)}\frac{E^{⁎}}{S^{⁎}}, \end{array}$$
  which implies that for the stability of interior equilibrium, the average cumulated time spent by individuals in the self-employed class and time in generating new vacancies by them is greater than or equal to the weighted average time require to fill the available vacancies.

## 7 The optimal control problem

Here, we present an optimal strategy for addressing unemployment within the informal sector by emphasizing on enhancing the success rate in the job search process and encouraging the self-employment by supporting informal workers in establishing their own businesses. To analyze this, we extend model system (1) by introducing two control variables u(t) and w(t) that minimizes $\overset{ˆ}{J}(u,w)$, written as follows:

(18) $$\overset{ˆ}{J}(u,w)=\int_{0}^{\overset{ˆ}{T}}[\eta_{1}U+\eta_{2}u^{2}(t)+\eta_{3}w^{2}(t)]dt$$

subject to

(19) $$\left\{ \begin{array}{cc} \frac{dU}{dt} & =A-k(1+u)UV+\theta E+\nu S-dU,\\ \frac{dE}{dt} & =k(1+u)UV-(\theta +\alpha +d)E-(r+w)E-\gamma SE,\\ \frac{dS}{dt} & =(r+w)E+\gamma SE-\nu S-dS,\\ \frac{dV}{dt} & =\phi S+(\alpha +d)E-\delta V-k(1+u)UV, \end{array}\right.$$

with $U(0)=U_{0}>0$, $E(0)=E_{0}\geq 0$, $S(0)=S_{0}\geq 0$ and $V(0)=V_{0}\geq 0$, as initial conditions. Here, $\overset{ˆ}{J}(u,w)$ represents the overall cost associated with the execution of proposed strategy aimed to reduce the unemployment over fixed time interval $\left[0,\;\overset{ˆ}{T}\right]$. In this context, $\eta_{1}$, $\eta_{2}$ and $\eta_{3}$ serve as weight constants. Specifically, $\eta_{1}$ quantifies the relative cost in alleviating the unemployment, $\eta_{2}$ represents the amount of money allocated for the execution of government policies aimed to enhance employment rate of job seekers and $\eta_{3}$ represents the amount of money spent on informal employees to support them in establishing their own businesses. The dynamical variables (U(t),E(t),S(t),V(t)) belong to the set of absolutely continuous functions from $\left[0,\;\overset{ˆ}{T}\right]$ to $R^{4}$. Further, u(t) represents the effectiveness of government policies in enhancing the employment rate of job seekers for the informal sector jobs and w(t) corresponds to the rate of informal workers for taking advantage of government initiatives framed to promote their self-employment for t≥0. Here, our goal is to obtain an optimal control pair $\left(u^{⁎},w^{⁎}\right)$ for $t\in [0,\overset{ˆ}{T}]$ in the set $U=\{(u(t),w(t))\in L^{1}(0,\overset{ˆ}{T})|(u(t),w(t))\in [0,\;u_{max}]\times [0,\;w_{max}]\;\forall \;t\in [0,\;\overset{ˆ}{T}]\}$, that minimizes the functional $\overset{ˆ}{J}(u,w)$ with respect to the system (19).

### 7.1 Existence of optimal control

The affirmation of the existence of optimal control can be derived by referring to the insights presented in the work of Fleming and Rishel [38]. The control problem can be written as

$$\min_{(u,w)\in U}\int_{0}^{\overset{ˆ}{T}}\overset{ˆ}{I}(\overset{˜}{X}(t),u(t),w(t))dt$$

subjected to

$$\begin{array}{cc} \overset{˙}{\overset{˜}{X}}(t)= & \overset{ˆ}{F}(\overset{˜}{X}(t),u(t),w(t)),a.e\;t\in [0,\;\overset{ˆ}{T}],\;\text{with}\;\overset{˜}{X}(0)={\overset{˜}{X}}_{0}=(U_{0},E_{0},S_{0},V_{0}), \end{array}$$

where $\overset{˜}{X}(t)=(U(t),E(t),S(t),V(t))\in AC([0,\;\overset{ˆ}{T}];R^{4})$, $\overset{ˆ}{I}(t)=\eta_{1}U+\eta_{2}u^{2}+\eta_{3}w^{2}$, and

$$\overset{ˆ}{F}(\overset{˜}{X},u,w)=\left(\begin{array}{c} A-k(1+u)UV+\theta E+\nu S-dU\\ k(1+u)UV-(\theta +\alpha +d)E-(r+w)E-\gamma SE\\ (r+w)E+\gamma SE-\nu S-dS\\ \phi S+(\alpha +d)E-\delta V-k(1+u)UV \end{array}\right).$$

The continuity of $\overset{ˆ}{I}$ and $\overset{ˆ}{F}$ with respect to $\overset{˜}{X}$, *u*, and *w* is evident and $\left(\overset{˜}{X}(t),\;u(t),\;w(t)\right)$ can be a feasible solution if it satisfies (19). According to the Fleming and Rishel's Theorem, the non-trivial requirements for the existence of an optimal solution of the proposed control problem are as follows:

(i) The set of all solutions of the system (19) with the control variables (u,w)∈U is non-empty.
(ii) The control set U is closed and convex. The vector function $\overset{ˆ}{F}(\overset{˜}{X},u,w)$ can be expressed as a linear function of the control variables with the coefficients depending on time and the dynamical variables.
(iii) The integrand of equation (18) is convex on the set U and additionally satisfies $\overset{ˆ}{I}(\overset{˜}{X},u,w)\geq i_{0}{\left\|(u,w)\right\|}^{i_{1}}-i_{2}$, where $i_{0}$ and $i_{1}>0$ and the ‖.‖ is a norm in $R^{2}$.

Applying the approach outlined in [39], we assert the subsequent theorem concerning the existence of an optimal solution for the control problem (18)-(19).

Theorem 7 *An optimal control pair*$\left(u^{⁎},\;w^{⁎}\right)$*within the set*U*exists and it serves to minimize the objective functional*(18)*with respect to the system*(19)*on the interval*$\left[0,\overset{ˆ}{T}\right]$*.*

**Proof:** The boundedness of the system (19) ensures the existence of its solutions, thus the set of controls and corresponding state variables are non-empty and it proves the condition (i). According to the definition of set U, it is evident that it is a closed and convex. Further, we can rewrite the function $\overset{ˆ}{F}(\overset{˜}{X},u,w)$ as

$$\begin{array}{cc} \overset{ˆ}{F}(\overset{˜}{X},u,w)= & \left[\begin{array}{c} A-kUV+\theta E+\nu S-dU\\ k(1+u)UV-(\theta +\alpha +d)E-(r+w)E-\gamma SE\\ (r+w)E+\gamma SE-(\nu +d)S\\ \phi S+(\alpha +d)E-\delta V-k(1+u)UV. \end{array}\right]+\left[\begin{array}{c} -kUV\\ kUV\\ 0\\ -kUV \end{array}\right]u+\left[\begin{array}{c} 0\\ -E\\ E\\ 0 \end{array}\right]w,\\ = & {\overset{ˆ}{f}}_{1}(t,\overset{˜}{X})+{\overset{ˆ}{f}}_{2}(t,\overset{˜}{X})u+{\overset{ˆ}{f}}_{3}(t,\overset{˜}{X})w. \end{array}$$

Therefore, condition (ii) holds. Further, we may note that $\overset{ˆ}{I}$ is a convex function on set U. Moreover, we have

$$\overset{ˆ}{I}(\overset{˜}{X},u,w)=\eta_{1}U+\eta_{2}u^{2}+\eta_{3}w^{2}\geq \frac{1}{2}\min(\eta_{2},\eta_{3}){\left\|(u,w)\right\|}^{2},$$

where $\|(u,w)\|=\sqrt{u^{2}+w^{2}}$ is the Euclidean norm in $R^{2}$, thus the condition (iii) is verified. Since all essential requirements for the existence of optimal solutions are satisfied. Therefore, a control pair $\left(u^{⁎},w^{⁎}\right)$ and corresponding optimal trajectory exist for the proposed control problem. Further, the uniqueness of the control solution can be proved by following the similar method given in [40].

### 7.2 Characterization of optimal control

After establishing the concept of optimal control, we apply Pontryagin's Principal [41], [42] to derive the essential conditions for optimal control. The Hamiltonian for the control problem can be expressed as

(20) $$\begin{array}{cc} \overset{˜}{H}(U,E,S,V,u,w,p_{1},p_{2},p_{3},p_{4})= & \eta_{1}U+\eta_{2}u^{2}(t)+\eta_{3}w^{2}(t)\\ & +p_{1}\left[A-k(1+u)UV+\theta E+\nu S-dU\right]\\ & +p_{2}\left[k(1+u)UV-(\theta +\alpha +d)E-(r+w)E-\gamma SE\right]\\ & +p_{3}\left[(r+w)E+\gamma SE-\nu S-dS\right]\\ & +p_{4}\left[\phi S+(\alpha +d)E-\delta V-k(1+u)UV\right]. \end{array}$$

In this context, $p_{j}$ (j=1,2,3,4) denotes the adjoint variables, and their corresponding differential equations can be described as follows:

(21) $$\begin{array}{cc} p_{1}^{\prime }= & -\frac{\partial \overset{˜}{H}}{\partial U}=-[\eta_{1}-p_{1}k(1+u)V-p_{1}d+p_{2}k(1+u)V-p_{4}k(1+u)V],\\ p_{2}^{\prime }= & -\frac{\partial \overset{˜}{H}}{\partial E}=-[p_{1}\theta -p_{2}(\theta +\alpha +d)-p_{2}(r+w+\gamma S)+p_{3}(r+w+\gamma S)+p_{4}(\alpha +d)],\\ p_{3}^{\prime }= & -\frac{\partial \overset{˜}{H}}{\partial S}=-[p_{1}\nu -p_{2}\gamma E+p_{3}\gamma E-p_{3}(\nu +d)+p_{4}\phi ],\\ p_{4}^{\prime }= & -\frac{\partial \overset{˜}{H}}{\partial V}=-[-p_{1}k(1+u)U+p_{2}k(1+u)U-p_{4}\delta -p_{4}k(1+u)U]. \end{array}$$

The transversality conditions can be expressed as $p_{j}(T)=0$, where j=(1,2,3,4). The optimal control pair, denoted as $\left(u^{⁎},\;w^{⁎}\right)$ can be obtained by using optimality conditions $\frac{\partial \overset{˜}{H}}{\partial u}=0$ and $\frac{\partial \overset{˜}{H}}{\partial w}=0$ in the required set U. Thus, we obtain $u^{⁎}=\frac{(p_{1}-p_{2}+p_{4})kU^{⁎}V^{⁎}}{2\eta_{2}}$ and $w^{⁎}=\frac{(p_{2}-p_{3})E^{⁎}}{2\eta_{3}}$ on the interior of the set U. By applying the bound constraints to the control, we derive the optimal control pair $\left(u^{⁎}(t),w^{⁎}(t)\right)$ as follows:

(22) $$u^{⁎}(t)=\min\left\{\max\left\{0,\frac{(p_{1}-p_{2}+p_{4})kU^{⁎}V^{⁎}}{2\eta_{2}}\right\},u_{max}\right\},$$

(23) $$w^{⁎}(t)=\min\left\{\max\left\{0,\frac{(p_{2}-p_{3})E^{⁎}}{2\eta_{3}}\right\},w_{max}\right\}.$$

Now, we summarize the result as follows:

Theorem 8 *The optimal control pair*$\left(u^{⁎},w^{⁎}\right)$*that minimizes the objective functional*(18)*with respect to the system*(19)*is defined by*(22)*-*(23)*.*

## 8 Numerical simulation

**Figure 4:**
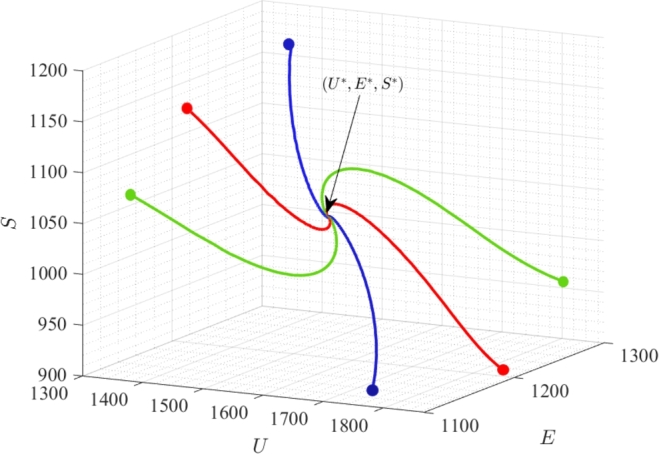
Global stability of *W*^⁎^ in *U* − *E* − *S* space.

In this section, we provide numerical validations of our analytically obtained results. For this, we choose a set of parameter values mentioned in Table 1, and maintain the consistency with this specific set of parameters throughout our simulations, unless explicitly specified. For parameter values given in Table 1, the value of reproduction number is obtained as $R_{0}=1.4286$ and the components of interior equilibrium $W^{⁎}$ are $U^{⁎}\approx 1521,\;\;E^{⁎}\approx 1225,\;\;S^{⁎}\approx 1026,\;\;V^{⁎}\approx 864$. The eigenvalues of the Jacobian matrix *J* at $W^{⁎}$ are determined as: −0.1016,−0.2526,−0.0109+0.0058i, and −0.0109−0.0058i. We note that the real parts of all eigenvalues are negative, affirming the local stability of $W^{⁎}$. Further, we plot the solution trajectories of system (1) originating from various initial states in the U−E−S space (Fig. 4). It becomes apparent that all these trajectories converge to the interior equilibrium $\left(U^{⁎},E^{⁎},S^{⁎}\right)$. This observation reasserts the global stability of equilibrium $W^{⁎}$ in U−E−S space.

**Table 1:** Description of parameters and their respective values.

Parameter	Description	Value
*A*	Rate of joining the unemployed class	50
*k*	Employment rate of unemployed individuals	0.00007
*θ*	Job expelling rate of employed individuals due to financial constraints	0.03
*ν*	Rate of leaving self-employment due to financial constraints	0.02
*α*	Resignation rate of employed individuals	0.01
*r*	Self-employment rate of employed individuals	0.02
*γ*	Movement rate of individuals from class *E* to *S* due to influence of self-employed peers	0.000005
*d*	Exit rate of individuals from each class due to natural mortality and emigration	0.01
*ϕ*	Rate of generating new jobs by self-employed individuals	0.15
*δ*	Reduction rate of newly generated jobs due to financial constraints	0.1

### 8.1 Sensitivity analysis

**Figure 5:**
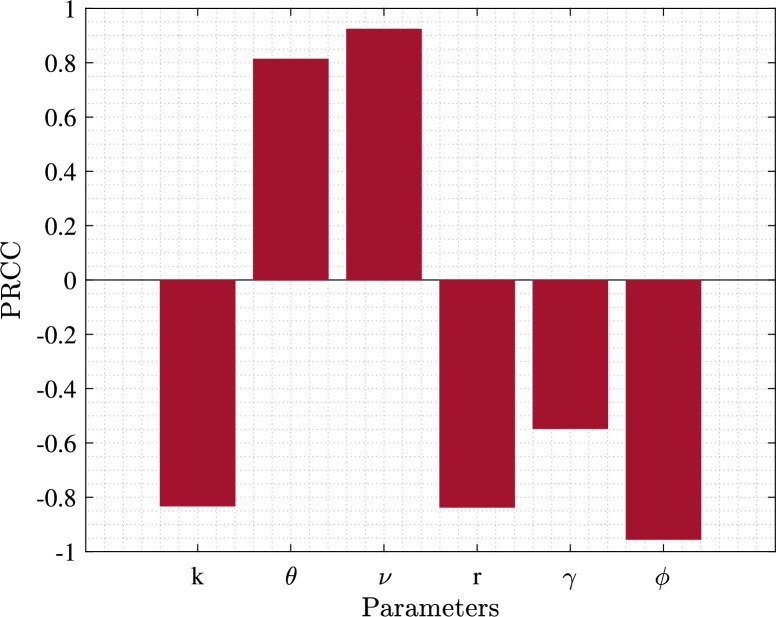
Effects of different parameters on number of unemployed individuals.

Sensitivity analysis is a useful tool for measuring uncertainty in complex mathematical models. It helps in identifying the crucial inputs (parameters) of a mathematical model and comprehending how uncertainty around these inputs influences the model's outcomes. A sensitivity analysis is considered to be global when all the input factors are varied simultaneously and the sensitivity is evaluated over the entire range of each input factor. Here, we perform a global sensitivity analysis to assess how model parameters influence the value of dynamical variables. To accomplish this, we apply two statistical approaches that are Latin Hypercube Sampling (LHS) and Partial Rank Correlation Coefficient (PRCC) described in [43], [44]. The PRCC vindicates to establish a relation between input parameters and the response function, while LHS enables the simultaneous variation of multiple parameters. The parameter values used for the simulations of the formulated system (1) may contain inaccuracies. However, applying PRCCs helps in mitigating the uncertainty associated with the selection of parameter values. Calculating the PRCC value offers benefits, as its sign indicates the nature of relationship and the magnitude represents the strength of correlations between the input parameters and model's output. In our analysis, we consider six input parameters: *k*, *θ*, *ν*, *r*, *γ*, and *ϕ*, with the number of unemployed individuals (*U*) as the output variable. This approach allows us to identify the correct correlation of these key parameters with the number of unemployed individuals, which is crucial for our studies. First, we confirm that there exists a monotone relation between input parameters and output function, as it is prerequisite for applying PRCC. To obtain sensitivity results, we use the baseline parameters' value listed in Table 1. Additionally, we introduce a ±25% deviation from the nominal values of each input parameter and consider a uniform distribution. We conduct 1000 simulations using Latin Hypercube Sampling (LHS) and subsequently compute the PRCC values and present them as a bar graph, Fig. 5. This figure illustrates that the parameters *θ* and *ν* have positive correlation with the number of unemployed individuals. Conversely, the parameters *k*, *r*, *γ*, and *ϕ* display negative correlations with the number of unemployed individuals. Furthermore, the figure highlights that among these parameters, *k*, *r*, *γ* and *ϕ* exert the most significant effect in reducing the number of unemployed individuals. This suggests that increasing the employment rate, self-employment rate, influence rate of self-employed individuals on employed individuals and the rate of generating jobs in the informal sector can effectively alleviate unemployment in developing countries.

### 8.2 Effects of some key parameters on equilibrium level of dynamical variables

**Figure 6:**
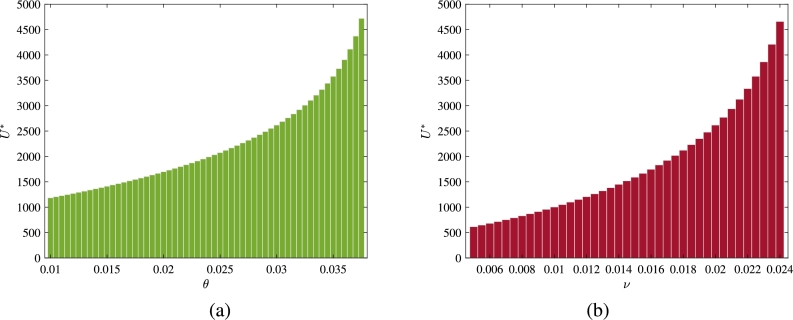
Effect of (a) job expelling rate of employed individuals (*θ*) and (b) rate of leaving self-employment (*ν*) on equilibrium level of unemployed individuals.

In the above subsection, through the approach of global sensitivity analysis, we have observed that the job firing rate of employed individuals (*θ*) and the leaving rate of self-employment due to financial constraints (*ν*) have a positive correlation with the number of unemployed individuals. Now, we plot bar diagrams to show the effects of parameters *θ* and *ν* on the equilibrium level of unemployed individuals (Fig. 6). These figures demonstrate that higher values of these two parameters positively affect the equilibrium level of $U^{⁎}$. Also, for the parameters' values given in Table 1, it becomes apparent that the leaving rate of self-employment has a more significant effect in raising the equilibrium level of $U^{⁎}$ compared to the job firing rate of employed individuals.

**Figure 7:**
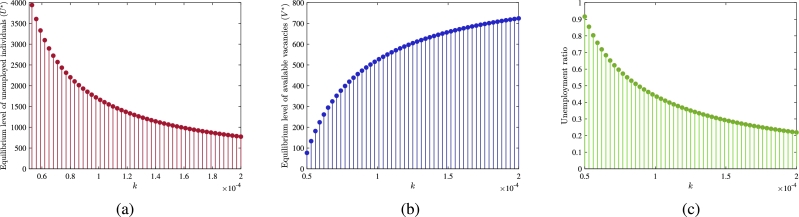
Effect of employment rate (*k*) (a) on the equilibrium level of unemployed individuals (b) on the equilibrium level of vacancies and (c) ratio of unemployed individuals in total considered workforce.

Furthermore, we plot the equilibrium level of unemployed individuals ($U^{⁎}$) and vacancies ($V^{⁎}$) by varying the employment rate of unemployed individuals (*k*) (Fig. 7(a)-(b)). These figures depict that with the rise in the employment rate, the level of $U^{⁎}$ decreases while the level of $V^{⁎}$ increases. This observation indicates that when a large number of unemployed individuals get employment in the informal sector, then it alleviates unemployment. On the other hand, the number of employed individuals increases, and their continuous shift towards self-employment increases the number of self-employed individuals who generate more job opportunities in the informal sector. Since the number of unemployed individuals decreases in the community, most generated vacancies remain vacant, increasing the equilibrium level of available vacancies. Moreover, these factors contribute to reducing the ratio of unemployed individuals ($U^{⁎}/(U^{⁎}+E^{⁎}+S^{⁎})$) in the total workforce (see Fig. 7(c)).

**Figure 8:**
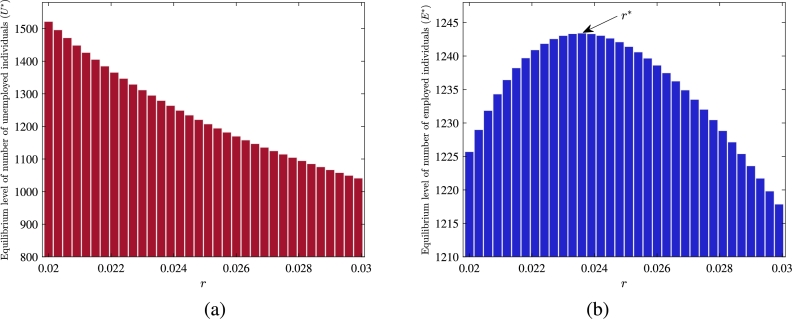
Variation in equilibrium components *U*^⁎^ and *E*^⁎^ with respect to *r*.

Now, we plot the equilibrium level of unemployed individuals ($U^{⁎}$) and number of employed individuals ($E^{⁎}$) by varying the self-transition rate of employed individuals towards self-employment (*r*) (Fig. 8 (a)-(b)). These figures depict that if the self-transition of employed individuals towards self-employment increases, the level of $U^{⁎}$ decreases (Fig. 8(a)). Furthermore, we get a threshold value of *r* (say $r^{⁎}$), such that for the values $r<r^{⁎}$, level of $E^{⁎}$ increases and for the values of $r>r^{⁎}$, the level of $E^{⁎}$ decreases (Fig. 8(b)). This dynamic is driven by the fact that when employed individuals of the informal sector shift towards self-employment and generate vacancies, it increases the number of vacancies available in the informal sector and reduces the number of unemployed individuals. Further, the number of employed individuals increases in the informal sector only when the rate of their self-shifting towards self-employment is less than a threshold value.

### 8.3 Effect of some parameters on $R_{0}$

**Figure 9:**
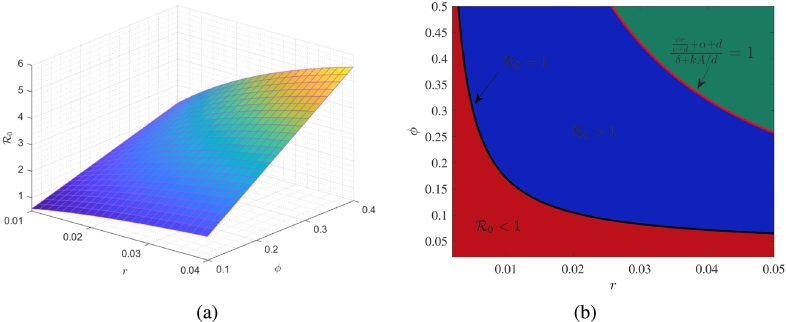
(a) Cumulative effect of *ϕ* and *r* on $R_{0}$. (b) Effect of *r* and *ϕ* on vacancy generation number $R_{v}$ and reproduction number $R_{0}$.

We have observed that the qualitative behavior of the proposed system (1) is affected by the value of reproduction number ($R_{0}$). Therefore, we analyze the effects of some key parameters on $R_{0}$. For this, we draw surface plot, representing $R_{0}$ in $\phi -r-R_{0}$ space by varying self-employment rate (*r*) and vacancy generation rate (*ϕ*) simultaneously (Fig. 9(a)). This figure illustrates that when *r* and *ϕ* have lower values, $R_{0}$ is also low. Conversely, as the values of these parameters increase, the values of $R_{0}$ increase. Socially, it indicates that when a large number of employed individuals willingly shift towards self-employment and generate a large number of vacancies, then the average number of vacancies increases, and due to this, the average number of employed individuals increases in the informal sector.

Previously, we have defined the vacancy generation ratio ‘$\left(\phi r/(\nu +d)+\alpha +d)/(\delta +kA/d)=R_{v}\;(say\right)$’, which represents the average number of vacancies generated in the informal sector. Thus, to analyze the relation among vacancies generation ratio ($R_{v}$) and reproduction ratio ($R_{0}$) affected due to variation of parameters *r* and *ϕ*, we plot the contours, representing the value of associated ratios equal to one, in ϕ−r plane (Fig. 9(b)). The black curve represents $R_{0}=1$ and red curve represents $R_{v}=1$. For each curve, the region lies above the curve represents the values of respective ratios greater than one, and the region below the curve represents the values of the corresponding ratios less than one. This diagram depicts that the value of $R_{v}$ may greater than one (green region) or less than one (blue region) for those values of *r* and *ϕ* where $R_{0}>1$. Additionally, for those values of *r* and *ϕ* where $R_{0}<1$ (red region), the $R_{v}$ is always less than one. Here, the blue region provides those values of the pair (r,ϕ) at which the reduction of unemployment is possible even when the average number of vacancies generated by each self-employed individual is less than one. This indicates that if a large number of informal employees willingly shift towards self-employment, it effectively reduces the number of unemployed individuals in the community, even when the job generation rate of self-employed individuals is very low.

**Figure 10:**
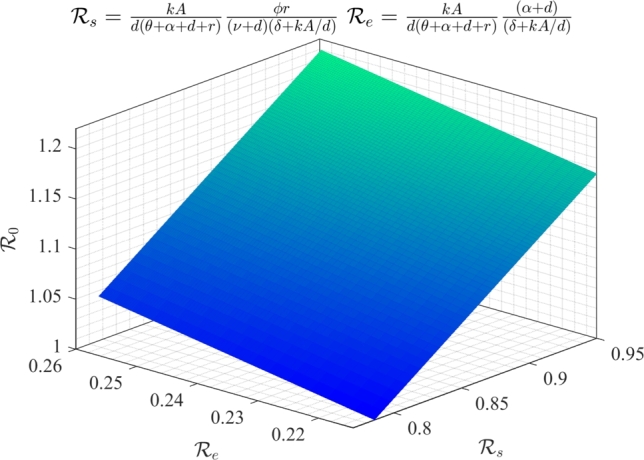
Surface plot for $R_{0}$ showing the cumulative effect of its two components $R_{s}$ and $R_{e}$ obtained by the variation of *k*.

Further, we generate a surface plot illustrating the reproduction number $R_{0}=R_{s}+R_{e}$ by varying the parameter *k* (Fig. 10). This figure depicts the cumulative effects of components $R_{s}$ and $R_{e}$, on $R_{0}$. Here, $R_{s}$ represents the average number of individuals who have gained employment due to vacancies generated by a self-employed person, while $R_{e}$ represents the average number of individuals who have obtained employment at positions vacated by previously employed individuals due to resignation or natural death. This figure depicts that even when the values of $R_{s}$ and $R_{e}$ are individually less than unity, their cumulative value representing the net reproduction number $R_{0}$ may exceed unity. Furthermore, $R_{s}$ holds a larger share than $R_{e}$ in the net value of $R_{0}$. It indicates that many unemployed individuals obtain employment in the informal sector due to vacancies created by self-employed individuals rather than the vacancies resulting from resignations or natural deaths of employed individuals within the informal sector.

### 8.4 Bifurcation analysis

**Figure 11:**
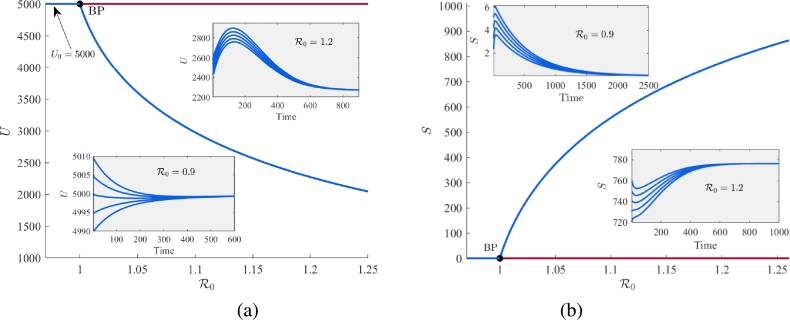
Equilibrium curve showing forward transcritical bifurcation in (a) $R_{0}-U$ plane and (b) $R_{0}-S$ plane.

**Figure 12:**
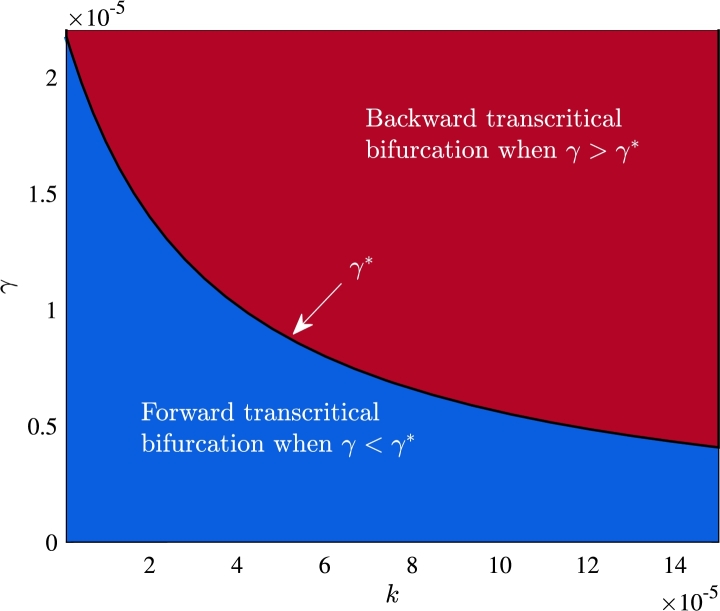
Region for forward and backward transcritical bifurcation in *k* − *γ* plane.

In this subsection, we provide the numerical validation of our analytically obtained results regarding different bifurcation phenomena. Now, we generate the equilibrium curve in $R_{0}-U$ and $R_{0}-S$ planes, shown in Fig. 11. In these figures, blue curve represents stable equilibrium and maroon curve represents unstable equilibrium. To instate variations in $R_{0}$, we vary the parameter *ϕ*, while maintaining the remaining parameter values constant as provided in Table 1. From these illustrations, it is clear that the formulated system (1) undergoes forward transcritical bifurcation at $R_{0}=1$ (equivalently, ϕ=0.105). Beyond this critical threshold, the stability of the *vacancy-free equilibrium* $W_{0}$ decimate leading to the stabilization of the interior equilibrium $W^{⁎}$. With increasing values of $R_{0}$, the equilibrium component corresponding to unemployed individuals decreases, while the component representing equilibrium level of self-employed individuals increases. It shows that when the values of $R_{0}<1$, the level of unemployment in the community is always high and conversely when the values of $R_{0}$ exceed one, the workforce experiences a low level of unemployment. Further, as we have analytically shown that the system (1) exhibits transcritical bifurcation in forward direction when $\gamma <\gamma^{⁎}$ and in backward direction when $\gamma >\gamma^{⁎}$ (Theorem 4). Now, we plot a curve (black color) for $\gamma^{⁎}$ in k−γ plane by varying the parameter *k* (Fig. 12). This curve divides the whole k−γ plane into two regions and at this curve, we can obtain the critical value of *γ* as $\gamma^{⁎}$ for a particular value of *k*. The region lies above the curve (maroon region) is for backward transcritical bifurcation, whereas the region below the curve (blue region) is for forward transcritical bifurcation. For the value of k=0.00007 and $R_{0}=1$ (or equivalently ϕ=0.105), we get $\gamma^{⁎}=0.00000723$.

**Figure 13:**
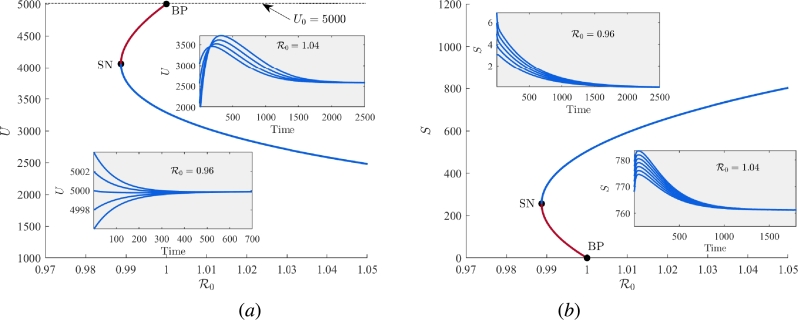
Equilibrium curve showing backward transcritical bifurcation in (*a*) $R_{0}-U$ plane and (*b*) $R_{0}-S$ plane.

Now, we set the value of $\gamma =0.00001>\gamma^{⁎}$ and plot the equilibrium curve again in the $R_{0}-U$ plane (Fig. 13(a)) and $R_{0}-S$ plane (Fig. 13(b)). We observe that in this scenario, the model system (1) undergoes transcritical bifurcation in backward direction along with the occurrence of saddle-node bifurcation at the point LP. Here, we can see that at the point LP, we get another critical value of $R_{0}$, say $R_{0}^{⁎}<1$, such that for $R_{0}<R_{0}^{⁎}$, interior equilibrium does not exist in the concerning plane, whereas for the value of $R_{0}^{⁎}<R_{0}<1$, one stable interior equilibrium $W_{1}^{⁎}$ exists along with stable *vacancy-free equilibrium* $W_{0}$. We observe that if $R_{0}>1$, solution trajectories approach to stable interior equilibrium and for $0<R_{0}<R_{0}^{⁎}$, solution trajectories approach to stable equilibrium $W_{0}$, at which the number of unemployed individuals is maximum. But for the range $R_{0}^{⁎}<R_{0}<1$, the system (1) shows bistable behavior and the convergence of solutions trajectories depends on initial conditions. If initially number of self-employed individuals is larger than the number of self-employed individuals at LP, solution trajectories converge to stable interior equilibrium with less number of unemployed individuals, on the contrary if initially the number of self-employed individuals is less in comparison to number of self-employed individuals at LP, then solutions trajectories converge to stable *vacancy-free equilibrium* with the maximum number of unemployed individuals.

**Figure 14:**
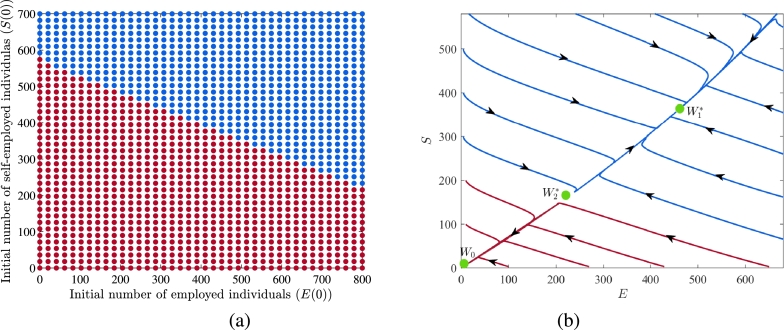
(a) Region of attraction showing two attractors in *E* − *S* plane for $R_{0}=0.995$ and (b) solution trajectories in *E* − *S* plane.

To further illustrate the bistable behavior of the system (1), we select a specific value of $R_{0}=0.995$ (or ϕ=0.1045) to generate the basin of attraction in the E−S plane, as shown in Fig. 14(a). In this figure, the blue dots represent the initial states of solution trajectories, which converge to the stable interior equilibrium $W_{1}^{⁎}$, while the maroon dots represent the initial states of solution trajectories that converge to the *vacancy-free equilibrium* $W_{0}$. The movement of solution trajectories with different initial states is depicted in Fig. 14(b). This bistable behavior of the system depicts that it is possible to reduce unemployment even when the value of $R_{0}$ is less than 1. However, achieving this goal requires the active participation of an adequate proportion of both employed and self-employed individuals within the informal sector.

### 8.5 Optimal control result

**Figure 15:**
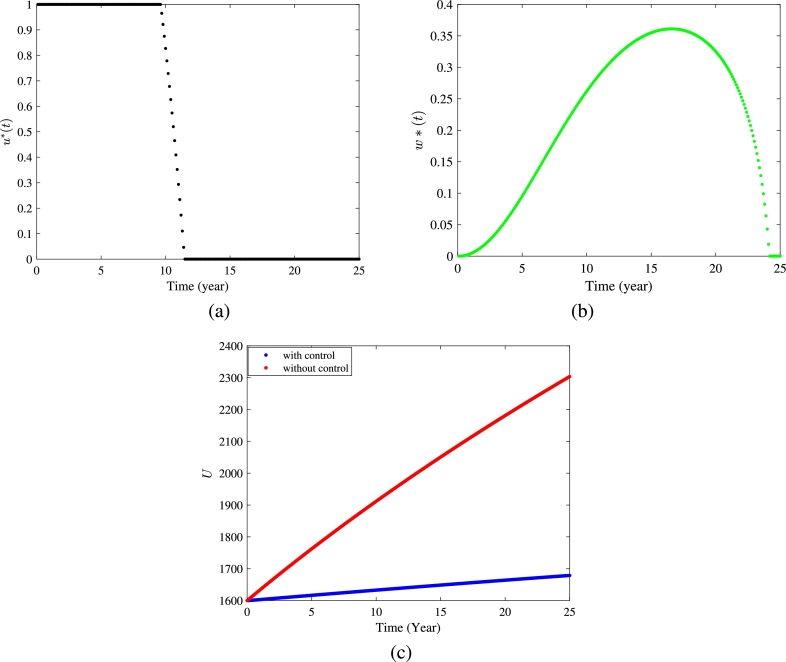
(a) Optimal control profile in *u*^⁎^, (b) optimal control profile in *w*^⁎^, and (c) variation in number of unemployed individuals with and without control strategy.

**Figure 16:**
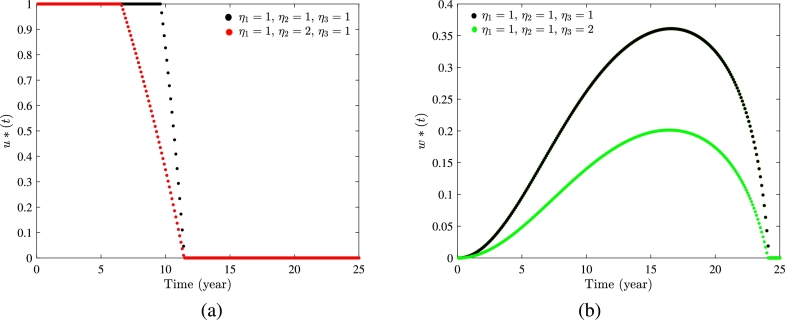
(a) Optimal control profile in *u*^⁎^, and (b) optimal control profile in *w*^⁎^, for different values of weight constants.

In this subsection, we apply a numerical approach forward-backward sweep method [45] to solve the optimal system. The procedure starts with an initial estimate of the control variables. We utilize a fourth-order Runge-Kutta iterative scheme to solve the system with dynamical variables forward in time, followed by solving the system with adjoint variables backward in time. These steps are repeated until the desired level of convergence is attained. For the optimization, we assign values to the weight factors $\eta_{1}=1$, $\eta_{2}=1$, and $\eta_{3}=1$ and set the final time $\overset{ˆ}{T}=25$ years. We fix minimum increment in control variable *u* at zero and the maximum increment at $u_{max}=1$. Similarly, we assign the minimum increment in control variable *w* at zero and maximum increment at $w_{max}=0.5$. The rest of the parameters involved in this procedure are same as in Table 1, except ϕ=0.12. We set the initial states for dynamical variables as (1600,200,90,70). The obtained optimal control profiles of $u^{⁎}$ and $w^{⁎}$ for 25 years are shown in Fig. 15(a) and Fig. 15(b), respectively. At each time step, the rate of getting employment by unemployed individuals is calculated as k(1+u) and rate of switching from informal employment to self-employment is calculated as r+w. From Fig. 15(a), we observe that to minimize the number of unemployed individuals in the time period of 25 years, the efficiency of government policies to enhance the success rate of unemployed individuals for securing employment should be maximized for the first 9.4 years. Further, for the following 2 years, this increment rate can be alleviated and after completing 11.5 years, there is no need to allocate funds to improve the success rate of unemployed individuals to secure employment. From the optimal control profile of the control variable *w* (Fig. 15 (b)), we observe that to promote the self-employment of informal workers during the fixed period of proposed strategy (i.e. 25 years), the government should progressively increase its initiatives for the initial 16 years, then there should be a sequential decrease over the subsequent 8 years. After applying the proposed optimal control strategy, a visible decrement in the number of unemployed individuals is evident, as shown in Fig. 15(c). For the given set of parameter values (Table 1), we observe an approximate 26% reduction in the number of unemployed individuals after implementing the control, at the end of the 25 years. Furthermore, if we change the values assigned to the weight constants, we obtain slightly different optimal profiles for the control variables under the proposed strategy, as shown in the Fig. 16.

## 9 Conclusion

In this article, a nonlinear mathematical model is proposed and analyzed to study the effect of informal sector employment on the unemployment dynamics of developing countries. A threshold quantity known as reproduction number ($R_{0}$) is determined. It is found that the proposed system has a *vacancy-free* equilibrium containing the maximum number of unemployed individuals, which remains stable when the value of reproduction number is less than one, and becomes unstable when the value of reproduction number exceeds unity. Apart from this, the proposed system has at most two interior equilibria, whose feasibility depends on the value of reproduction number. Sufficient conditions for the local and global stability of these interior equilibria have been determined. Further, it is observed that the system exhibits various dynamical behaviors, including transcritical (forward or backward) and saddle-node bifurcations, as the value of reproduction number varies.

Our analysis demonstrates that the proposed system exhibits transcritical bifurcation in the forward direction when the influence rate of self-employed community on informal employees for being self-employed is below a threshold value ($\gamma^{⁎}$), and the direction of bifurcation switches to backward if this influence rate surpasses its threshold value. The occurrence of transcritical bifurcation in the forward direction implies that the equilibrium level of unemployed individuals remains consistently high in the considered region when the reproduction number is less than unity. However, a reduction in the equilibrium level of unemployed individuals becomes achievable when the reproduction number exceeds unity. On the other hand, the occurrence of transcritical bifurcation in the backward direction implies that the reduction in the equilibrium level of unemployed individuals is possible even when the value of the reproduction number is less than one. However, achieving this goal requires a sufficient number of informal employees and self-employed individuals to be present in the informal sector.

Furthermore, through sensitivity analysis, we have determined that the employment rate (*k*), self-employment rate (*r*), and vacancy generation rate (*ϕ*) are the most crucial parameters in reducing the level of unemployment. Additionally, it is observed that a significant number of unemployed individuals obtain employment in the informal sector due to vacancies created by self-employed individuals rather than the vacancies resulting from resignations or natural deaths of employed individuals. Moreover, a substantial shift of informal workers towards self-employment proves to be highly effective in lowering the level of unemployment, even when each self-employed individual, on average, generates a modest number of vacancies. Therefore, it is imperative for the government of developing nations to promote self-regulated businesses within the informal sector as a aim to combat unemployment. Moreover, by employing optimal control theory, we have discussed an optimal strategy for implementing government policies in an optimized manner to improve the employment rate of unemployed individuals and promote self-employment among informal employees. This analysis underscores the need for an optimal approach to efficiently minimize expenses associated with enhancing the employability of unemployed individuals and promoting self-employment among informal employees, which ultimately contribute in reducing unemployment in developing countries.

## 10 Advantages, future directives, and limitations

The proposed model offers a qualitative understanding of the complex dynamics that exist between employees and self-employed individuals within informal sector, shedding light on its impact on unemployment. Moreover, it enables us to pinpoint critical parameters associated with this sector that effectively reduce unemployment. We have also discussed possible dynamic scenarios that can occur due to parameter variations. Furthermore, the obtained results are verified through numerical simulations using a hypothetical set of parameter values. These analytically obtained results can be further validated using real parameter values if the detailed data of informal sector is available. Therefore, this study can be extended from a data analysis perspective. Moreover, some time lags exist between the movement of informal employees towards self-employment and job generation. These time lags are not considered in the model formulation, however, it may affect the dynamics of considered variables in the formulated model system.

## Funding statement

Mohammad Sajid is supported by the Deanship of Graduate Studies and Scientific Research, 10.13039/501100007414Qassim University, Saudi Arabia for funding of APC (QU-APC-2024-9/1) for this publication.

## CRediT authorship contribution statement

**A.K. Misra:** Writing – review & editing, Methodology, Investigation, Conceptualization. **Mamta Kumari:** Writing – original draft, Methodology, Investigation, Formal analysis, Conceptualization. **Mohammad Sajid:** Writing – review & editing, Methodology, Funding acquisition, Conceptualization.

## Declaration of Competing Interest

The authors declare that they have no known competing financial interests or personal relationships that could have appeared to influence the work reported in this paper.

## Data Availability

Data included in article/supplementary material/referenced in article.

## Appendix A

Proof The Jacobian matrix $J_{W_{0}}$ has zero eigenvalue at $R_{0}=1$ and rest of the eigenvalues are negative. Thus, equilibrium $W_{0}$ is non-hyperbolic at $R_{0}=1$. To prove the occurrence of transcritical bifurcation at $R_{0}=1$, we analyze the sign of expressions $\overset{¯}{a}$ and $\overset{¯}{b}$ defined in [46]. For this, let $U={\overset{¯}{x}}_{1}$, $E={\overset{¯}{x}}_{2}$, $S={\overset{¯}{x}}_{3}$, and $V={\overset{¯}{x}}_{4}$ and considering $R_{0}$ (or equivalent $\phi =\phi_{c}$) as bifurcation parameter. Then, the right hand side of the equations defined in system (1) can be written as follows:

$$\begin{array}{cc} f_{1} & :=A-k{\overset{¯}{x}}_{1}{\overset{¯}{x}}_{4}+\theta {\overset{¯}{x}}_{2}+\nu {\overset{¯}{x}}_{3}-d{\overset{¯}{x}}_{1},\\ f_{2} & :=k{\overset{¯}{x}}_{1}{\overset{¯}{x}}_{4}-(\theta +\alpha +d){\overset{¯}{x}}_{2}-r{\overset{¯}{x}}_{2}-\gamma {\overset{¯}{x}}_{2}{\overset{¯}{x}}_{3},\\ f_{3} & :=r{\overset{¯}{x}}_{2}+\gamma {\overset{¯}{x}}_{2}{\overset{¯}{x}}_{3}-\nu {\overset{¯}{x}}_{3}-d{\overset{¯}{x}}_{3},\\ f_{4} & :=\phi {\overset{¯}{x}}_{3}+(\alpha +d){\overset{¯}{x}}_{2}-\delta {\overset{¯}{x}}_{4}-k{\overset{¯}{x}}_{1}{\overset{¯}{x}}_{4}. \end{array}$$

We obtain the right eigenvector $V={\left[{\overset{¯}{v}}_{1}\;{\overset{¯}{v}}_{2}\;{\overset{¯}{v}}_{3}\;{\overset{¯}{v}}_{4}\right]}^{⊺}$ and left eigenvector $W=[{\overset{¯}{w}}_{1}\;{\overset{¯}{w}}_{2}\;{\overset{¯}{w}}_{3}\;{\overset{¯}{w}}_{4}]$ corresponding to the zero eigenvalue of the Jacobian matrix $J_{W_{0}}$ as follows

$$V=\left[\begin{array}{c} -(rd+(\alpha +d)(\nu +d))kA/d\\ d(\nu +d)kA/d\\ rdkA/d\\ d(\theta +\alpha +d+r)(\nu +d) \end{array}\right],\;\;\;\;\text{and}\;\;\;\;W={\left[\begin{array}{c} 0\\ \left(\nu +d)(\delta +kA/d\right)\\ \phi kA/d\\ (\nu +d)kA/d \end{array}\right]}^{⊺}.$$

For the model system (1), constants $\overset{¯}{a}$ and $\overset{¯}{b}$ defined in Theorem 4.1 of [46] are given as

$$\overset{¯}{a}=\sum_{i,j,k=1}^{4}{\overset{¯}{w}}_{k}{\overset{¯}{v}}_{i}{\overset{¯}{v}}_{j}\frac{\partial^{2}f_{k}}{\partial {\overset{¯}{x}}_{i}\partial {\overset{¯}{x}}_{j}}(I_{0},\;\phi_{c})\;\;\;\;\text{and}\;\;\;\;\overset{¯}{b}=\sum_{k,i=1}^{4}{\overset{¯}{w}}_{k}{\overset{¯}{v}}_{i}\frac{\partial^{2}f_{k}}{\partial {\overset{¯}{x}}_{i}\partial \phi }(I_{0},\;\phi_{c}).$$

Here, ${\overset{¯}{v}}_{i}$ and ${\overset{¯}{w}}_{k}$ (i,k=1,2,3,4) are representing the components of right and left eigenvectors, respectively. Thus, we obtain

$$\begin{array}{cc} \overset{¯}{a} & =2k^{2}{\left(\nu +d\right)}^{2}A\left[\gamma A\left(\delta (\theta +\alpha +d)+\frac{\theta kA}{d}\right)-(\theta +\alpha +d)((\nu +d)(\alpha +d)+rd)\right]\\ & =2k^{2}{\left(\nu +d\right)}^{2}A^{2}\left(\delta (\theta +\alpha +d)+\frac{\theta kA}{d}\right)(\gamma -\gamma^{⁎}),\;\;\text{and}\\ \overset{¯}{b} & =r(\nu +d)k^{2}A^{2}/d>0, \end{array}$$

where $\gamma^{⁎}=((\theta +\alpha +d)((\alpha +d)(\nu +d)+rd))/(A(\delta (\theta +\alpha +d)+\theta kA/d))$. It can be observed that $\overset{¯}{b}>0$, while $\overset{¯}{a}$ is positive when $\gamma >\gamma^{⁎}$ and negative when $\gamma <\gamma^{⁎}$. Therefore, result concerning the occurrence of transcritical bifurcation is concluded.

## Appendix B

Proof Let $R_{0}^{⁎}$ (or equivalent $\phi_{c}^{⁎}$) be such that for $R_{0}^{⁎}<R_{0}<1$, system (1) has two branches of interior equilibria (for $W_{1}^{⁎}$ and $W_{2}^{⁎}$) and at $R_{0}=R_{0}^{⁎}$, these two branches of equilibria unite at a single interior equilibrium (i.e., $W_{3}^{⁎}$) and for $0<R_{0}<R_{0}^{⁎}$, the system (1) has no interior equilibrium. The Jacobian matrix at equilibrium $W_{3}^{⁎}=(U_{3}^{⁎},\;E_{3}^{⁎},\;S_{3}^{⁎},\;V_{3}^{⁎})$ is obtained as:

$$J{|}_{W_{3}^{⁎}}=\begin{array}{c} \left[\begin{array}{cccc} -{\overset{¯}{c}}_{11}\; & \theta \; & \nu \; & -kU_{3}^{⁎}\\ kV_{3}^{⁎}\; & -{\overset{¯}{c}}_{22}\; & -\gamma E_{3}^{⁎}\; & kU_{3}^{⁎}\\ 0\; & {\overset{¯}{c}}_{32}\; & -{\overset{¯}{c}}_{33}\; & 0\\ -kV_{3}^{⁎}\; & {\overset{¯}{c}}_{42}\; & \phi \; & -{\overset{¯}{c}}_{44} \end{array}\right], \end{array}$$

where ${\overset{¯}{c}}_{11}=d+kV_{3}^{⁎}$, ${\overset{¯}{c}}_{22}=\theta +\alpha +d+r+\gamma S_{3}^{⁎}$, ${\overset{¯}{c}}_{32}=r+\gamma S_{3}^{⁎}$, ${\overset{¯}{c}}_{33}=\nu +d-\gamma E_{3}^{⁎}$, ${\overset{¯}{c}}_{42}=\alpha +d$, and ${\overset{¯}{c}}_{44}=\delta +kU_{3}^{⁎}$. Thus, the corresponding characteristic equation is

(24) $$\psi^{4}+\overset{˜}{C_{1}}\psi^{3}+\overset{˜}{C_{2}}\psi^{2}+\overset{˜}{C_{3}}\psi +\overset{˜}{C_{4}}=0,$$

where

$$\begin{array}{cc} \overset{˜}{C_{1}}= & {\overset{¯}{c}}_{11}+{\overset{¯}{c}}_{22}+{\overset{¯}{c}}_{33}+{\overset{¯}{c}}_{44},\\ \overset{˜}{C_{2}}= & {\overset{¯}{c}}_{11}{\overset{¯}{c}}_{22}+{\overset{¯}{c}}_{11}{\overset{¯}{c}}_{33}+{\overset{¯}{c}}_{11}{\overset{¯}{c}}_{44}+{\overset{¯}{c}}_{22}{\overset{¯}{c}}_{33}+{\overset{¯}{c}}_{22}{\overset{¯}{c}}_{44}+{\overset{¯}{c}}_{33}{\overset{¯}{c}}_{44}-k^{2}U_{3}^{⁎}V_{3}^{⁎}-\theta kV_{3}^{⁎}+\gamma E_{3}^{⁎}{\overset{¯}{c}}_{32}-kU_{3}^{⁎}{\overset{¯}{c}}_{42},\\ \overset{˜}{C_{3}}= & {\overset{¯}{c}}_{11}{\overset{¯}{c}}_{22}{\overset{¯}{c}}_{33}+{\overset{¯}{c}}_{11}{\overset{¯}{c}}_{22}{\overset{¯}{c}}_{44}+{\overset{¯}{c}}_{11}{\overset{¯}{c}}_{33}{\overset{¯}{c}}_{44}+{\overset{¯}{c}}_{22}{\overset{¯}{c}}_{33}{\overset{¯}{c}}_{44}+k^{2}U_{3}^{⁎}V_{3}^{⁎}({\overset{¯}{c}}_{42}+\theta )+\gamma E_{3}^{⁎}{\overset{¯}{c}}_{32}({\overset{¯}{c}}_{11}+{\overset{¯}{c}}_{44})-kU_{3}^{⁎}(\phi {\overset{¯}{c}}_{32}+{\overset{¯}{c}}_{42}{\overset{¯}{c}}_{11}+{\overset{¯}{c}}_{42}{\overset{¯}{c}}_{22})\\ & -\theta kV_{3}^{⁎}({\overset{¯}{c}}_{33}+{\overset{¯}{c}}_{44})-k^{2}U_{3}^{⁎}V_{3}^{⁎}({\overset{¯}{c}}_{22}+{\overset{¯}{c}}_{33})-\nu kV_{3}^{⁎}{\overset{¯}{c}}_{32},\\ \overset{˜}{C_{4}}= & {\overset{¯}{c}}_{11}{\overset{¯}{c}}_{22}{\overset{¯}{c}}_{33}{\overset{¯}{c}}_{44}+\gamma E_{3}^{⁎}{\overset{¯}{c}}_{11}{\overset{¯}{c}}_{32}{\overset{¯}{c}}_{44}+k^{2}U_{3}^{⁎}V_{3}^{⁎}(\phi {\overset{¯}{c}}_{32}++\nu {\overset{¯}{c}}_{32}+\theta {\overset{¯}{c}}_{33}+{\overset{¯}{c}}_{42}{\overset{¯}{c}}_{33})-k^{2}U_{3}^{⁎}V_{3}^{⁎}({\overset{¯}{c}}_{22}{\overset{¯}{c}}_{33}+\gamma E_{3}^{⁎}{\overset{¯}{c}}_{32})\\ & -kV_{3}^{⁎}{\overset{¯}{c}}_{44}(\theta {\overset{¯}{c}}_{33}+\nu {\overset{¯}{c}}_{32})-kU_{3}^{⁎}{\overset{¯}{c}}_{11}(\phi {\overset{¯}{c}}_{32}+{\overset{¯}{c}}_{42}{\overset{¯}{c}}_{33}). \end{array}$$

It is clear that $\overset{˜}{C_{1}}$ and $\overset{˜}{C_{2}}$ are positive. If $\overset{˜}{C_{4}}=0$, then $\overset{˜}{C_{3}}>0$ and after some simplifications, we get $\overset{˜}{C_{1}}\overset{˜}{C_{2}}-\overset{˜}{C_{3}}>0$. Consequently, applying the *Routh-Hurwitz criterion*, we can conclude that characteristic equation (24) has a zero root and remaining roots are either negative or have negative real parts. Thus, equilibrium $W_{3}^{⁎}$ is non-hyperbolic. It is easy to obtain the left eigenvector say $\overset{ˆ}{W}=[w_{1}\;w_{2}\;w_{3}\;w_{4}]$ and right eigenvector say $\overset{ˆ}{V}={\left[v_{1}\;v_{2}\;v_{3}\;v_{4}\right]}^{⊺}$ corresponding to zero eigenvalue of the Jacobian matrix $J{|}_{W_{3}^{⁎}}$, where

$$\overset{ˆ}{V}=\left[\begin{array}{c} -(d{\overset{¯}{c}}_{32}+{\overset{¯}{c}}_{42}{\overset{¯}{c}}_{33})\delta \\ \delta d{\overset{¯}{c}}_{33}\\ \delta d{\overset{¯}{c}}_{32}\\ ({\overset{¯}{c}}_{44}-{\overset{¯}{c}}_{22}){\overset{¯}{c}}_{33}+(\phi -\gamma E_{3}^{⁎}){\overset{¯}{c}}_{32} \end{array}\right]\;\;\;\text{and}\;\;\;\;\overset{ˆ}{W}={\left[\begin{array}{c} \delta kV_{3}^{⁎}{\overset{¯}{c}}_{33}\\ (\delta kV_{3}^{⁎}+d{\overset{¯}{c}}_{44}){\overset{¯}{c}}_{33}\\ \nu \delta kV_{3}^{⁎}+\phi kdU_{3}^{⁎}-\gamma E_{3}^{⁎}(\delta kV_{3}^{⁎}+d{\overset{¯}{c}}_{44})\\ dkU_{3}^{⁎}{\overset{¯}{c}}_{33} \end{array}\right]}^{⊺}.$$

The model system (1) can be represented as $d\overset{ˆ}{X}/dt=\overset{ˆ}{G}(\overset{ˆ}{X})$, where $\overset{ˆ}{X}=(U,E,S,V)$ and $\overset{ˆ}{G}(\overset{ˆ}{X})=({\overset{ˆ}{G}}_{1}(\overset{ˆ}{X}),{\overset{ˆ}{G}}_{2}(\overset{ˆ}{X}),{\overset{ˆ}{G}}_{3}(\overset{ˆ}{X}),{\overset{ˆ}{G}}_{4}(\overset{ˆ}{X}))$. Thus, we get

$$\begin{array}{cc} \overset{ˆ}{S_{1}} & ={\overset{ˆ}{W}.\frac{d\overset{ˆ}{G}}{d\phi }|}_{\left(W_{3}^{⁎},\phi_{c}^{⁎}\right)}=dk{\overset{¯}{c}}_{33}U_{3}^{⁎}S_{3}^{⁎}>0,\;\;\text{and}\\ \overset{ˆ}{S_{2}} & ={\left[\overset{ˆ}{W}.D^{2}\overset{ˆ}{G}(\overset{ˆ}{V},\overset{ˆ}{V})\right]}_{\left(W_{3}^{⁎},\phi_{c}^{⁎}\right)}=2\{\gamma (w_{3}-w_{2})v_{2}v_{3}+kv_{1}v_{4}(w_{2}-w_{1}-w_{4})\}. \end{array}$$

The condition of Sotomayor's theorem for the existence of saddle-node bifurcation is satisfied if $S_{2}\neq 0$. Thus, a smooth equilibrium curve of model system (1) in $R^{4}\times R$ passing through $\left(W_{3}^{⁎},\phi_{c}^{⁎}\right)$ and tangent to the hyperplane $R^{4}\times \{\phi_{c}^{⁎}\}$. No equilibrium point exists, when $\phi <\phi_{c}^{⁎}$, whereas for $\phi >\phi_{c}^{⁎}$, two equilibria exist: one is a stable node and the other one is a saddle in nature.

## Appendix C

Proof Let L be a positive definite function defined as:

$$L=U^{⁎}\overset{˜}{g}\left(\frac{U}{U^{⁎}}\right)+l_{1}E^{⁎}\overset{˜}{g}\left(\frac{E}{E^{⁎}}\right)+l_{2}S^{⁎}\overset{˜}{g}\left(\frac{S}{S^{⁎}}\right)+l_{3}V^{⁎}\overset{˜}{g}\left(\frac{V}{V^{⁎}}\right),$$

where $l_{1}$, $l_{2}$ and $l_{3}$ are positive constants, to be chosen appropriately and $\overset{˜}{g}(x)=x-1-\ln x$ is Volterra-type Lyapunov function. The function $\overset{˜}{g}$ has a global minimum at x=1, such that $\overset{˜}{g}(x)>\overset{˜}{g}(1)$ ∀ x>0 and the equality holds at x=1 only [47]. Taking the time derivative of L along the solutions of the system (1), we obtain the following:

$$\begin{array}{cc} \frac{dL}{dt} & =\left(1-\frac{U^{⁎}}{U}\right)\overset{˙}{U}+l_{1}\left(1-\frac{E^{⁎}}{E}\right)\overset{˙}{E}+l_{2}\left(1-\frac{S^{⁎}}{S}\right)\overset{˙}{S}+l_{3}\left(1-\frac{V^{⁎}}{V}\right)\overset{˙}{V}\\ & =\left(1-\frac{U^{⁎}}{U}\right)\left[A\left(1-\frac{U}{U^{⁎}}\right)-kU^{⁎}V^{⁎}\left(\frac{UV}{U^{⁎}V^{⁎}}-{\frac{U}{U}}^{⁎}\right)+\theta E^{⁎}\left(\frac{E}{E^{⁎}}-\frac{U}{U^{⁎}}\right)+\nu S^{⁎}\left(\frac{S}{S^{⁎}}-\frac{U}{U^{⁎}}\right)\right]\\ & +l_{1}\left(1-\frac{E^{⁎}}{E}\right)\left[kU^{⁎}V^{⁎}\left(\frac{UV}{U^{⁎}V^{⁎}}-\frac{E}{E^{⁎}}\right)-\gamma S^{⁎}E^{⁎}\left(\frac{ES}{E^{⁎}S^{⁎}}-\frac{E}{E^{⁎}}\right)\right]\\ & +l_{2}\left(1-\frac{S^{⁎}}{S}\right)\left[rE^{⁎}\left(\frac{E}{E^{⁎}}-\frac{S}{S^{⁎}}\right)+\gamma S^{⁎}E^{⁎}\left(\frac{ES}{E^{⁎}S^{⁎}}-\frac{S}{S^{⁎}}\right)\right]\\ & +l_{3}\left(1-\frac{V^{⁎}}{V}\right)\left[\phi S^{⁎}\left(\frac{S}{S^{⁎}}-\frac{V}{V^{⁎}}\right)+(\alpha +d)E^{⁎}\left(\frac{E}{E^{⁎}}-\frac{V}{V^{⁎}}\right)-kU^{⁎}V^{⁎}\left(\frac{UV}{U^{⁎}V^{⁎}}-\frac{V}{V^{⁎}}\right)\right]. \end{array}$$

Expanding the above expression, we get the following

$$\begin{array}{cc} \frac{dL}{dt}= & A\left(2-\frac{U}{U^{⁎}}-\frac{U^{⁎}}{U}\right)-kU^{⁎}V^{⁎}\left(\frac{UV}{U^{⁎}V^{⁎}}-\frac{U}{U^{⁎}}-\frac{V}{V^{⁎}}+1\right)+\theta E^{⁎}\left(\frac{E}{E^{⁎}}-\frac{U}{U^{⁎}}-\frac{EU^{⁎}}{E^{⁎}U}+1\right)\\ & +\nu S^{⁎}\left(\frac{S}{S^{⁎}}-\frac{U}{U^{⁎}}-\frac{SU^{⁎}}{S^{⁎}U}+1\right)+l_{1}kU^{⁎}V^{⁎}\left(\frac{UV}{U^{⁎}V^{⁎}}-\frac{E}{E^{⁎}}-\frac{UVE^{⁎}}{U^{⁎}V^{⁎}E}+1\right)-l_{1}\gamma S^{⁎}E^{⁎}\left(\frac{ES}{E^{⁎}S^{⁎}}-\frac{S}{S^{⁎}}-\frac{E}{E^{⁎}}+1\right)\\ & +l_{2}rE^{⁎}\left(\frac{E}{E^{⁎}}-\frac{S}{S^{⁎}}-\frac{S^{⁎}E}{SE^{⁎}}+1\right)+l_{2}\gamma S^{⁎}E^{⁎}\left(\frac{ES}{E^{⁎}S^{⁎}}-\frac{S}{S^{⁎}}-\frac{E}{E^{⁎}}+1\right)+l_{3}\phi S^{⁎}\left(\frac{S}{S^{⁎}}-\frac{V}{V^{⁎}}-\frac{V^{⁎}S}{VS^{⁎}}+1\right)\\ & +l_{3}(\alpha +d)E^{⁎}\left(\frac{E}{E^{⁎}}-\frac{V}{V^{⁎}}-\frac{V^{⁎}E}{VE^{⁎}}+1\right)-l_{3}kU^{⁎}V^{⁎}\left(\frac{UV}{U^{⁎}V^{⁎}}-\frac{U}{U^{⁎}}-\frac{V}{V^{⁎}}+1\right). \end{array}$$

We can rewrite the above expression as:

$$\begin{array}{cc} \frac{dL}{dt}= & A\left[-\overset{˜}{g}\left(\frac{U}{U^{⁎}}\right)-\overset{˜}{g}\left(\frac{U^{⁎}}{U}\right)\right]-kU^{⁎}V^{⁎}\left[\overset{˜}{g}\left(\frac{UV}{U^{⁎}V^{⁎}}\right)-\overset{˜}{g}\left(\frac{U}{U^{⁎}}\right)-\overset{˜}{g}\left(\frac{V}{V^{⁎}}\right)\right]+\theta E^{⁎}\left[\overset{˜}{g}\left(\frac{E}{E^{⁎}}\right)-\overset{˜}{g}\left(\frac{U}{U^{⁎}}\right)-\overset{˜}{g}\left(\frac{EU^{⁎}}{E^{⁎}U}\right)\right]\\ & +\nu S^{⁎}\left[\overset{˜}{g}\left(\frac{S}{S^{⁎}}\right)-\overset{˜}{g}\left(\frac{U}{U^{⁎}}\right)-\overset{˜}{g}\left(\frac{SU^{⁎}}{S^{⁎}U}\right)\right]+l_{1}kU^{⁎}V^{⁎}\left[\overset{˜}{g}\left(\frac{UV}{U^{⁎}V^{⁎}}\right)-\overset{˜}{g}\left(\frac{E}{E^{⁎}}\right)-\overset{˜}{g}\left(\frac{UVE^{⁎}}{U^{⁎}V^{⁎}E}\right)\right]\\ & -l_{1}\gamma S^{⁎}E^{⁎}\left[\overset{˜}{g}\left(\frac{ES}{E^{⁎}S^{⁎}}\right)-\overset{˜}{g}\left(\frac{E}{E^{⁎}}\right)-\overset{˜}{g}\left(\frac{S}{S^{⁎}}\right)\right]+l_{2}rE^{⁎}\left[\overset{˜}{g}\left(\frac{E}{E^{⁎}}\right)-\overset{˜}{g}\left(\frac{S}{S^{⁎}}\right)-\overset{˜}{g}\left(\frac{ES^{⁎}}{E^{⁎}S}\right)\right]\\ & +l_{2}\gamma S^{⁎}E^{⁎}\left[\overset{˜}{g}\left(\frac{ES}{E^{⁎}S^{⁎}}\right)-\overset{˜}{g}\left(\frac{E}{E^{⁎}}\right)-\overset{˜}{g}\left(\frac{S}{S^{⁎}}\right)\right]+l_{3}\phi S^{⁎}\left[\overset{˜}{g}\left(\frac{S}{S^{⁎}}\right)-\overset{˜}{g}\left(\frac{V}{V^{⁎}}\right)-\overset{˜}{g}\left(\frac{SV^{⁎}}{S^{⁎}V}\right)\right]\\ & l_{3}(\alpha +d)E^{⁎}\left[\overset{˜}{g}\left(\frac{E}{E^{⁎}}\right)-\overset{˜}{g}\left(\frac{V}{V^{⁎}}\right)-\overset{˜}{g}\left(\frac{EV^{⁎}}{E^{⁎}V}\right)\right]-l_{3}kU^{⁎}V^{⁎}\left[\overset{˜}{g}\left(\frac{UV}{U^{⁎}V^{⁎}}\right)-\overset{˜}{g}\left(\frac{U}{U^{⁎}}\right)-\overset{˜}{g}\left(\frac{V}{v^{⁎}}\right)\right]. \end{array}$$

Choosing $l_{1}=1+\frac{kU^{⁎}}{\delta }$, $l_{2}=l_{1}$, and $l_{3}=\frac{kU^{⁎}}{\delta }$ and using the equations (3)-(6), the above expression can be reduced as follows:

(25) $$\begin{array}{cc} \frac{dL}{dt}= & \left(-dU^{⁎}+\frac{kU^{⁎}}{\delta }kU^{⁎}V^{⁎}\right)\overset{˜}{g}\left(\frac{U}{U^{⁎}}\right)-A\overset{˜}{g}\left(\frac{U}{U^{⁎}}\right)-\left((\alpha +d)E^{⁎}+\gamma S^{⁎}E^{⁎}+\frac{kU^{⁎}}{\delta }(\theta +\gamma S^{⁎}E^{⁎})\right)\overset{˜}{g}\left(\frac{E}{E^{⁎}}\right)\\ & +\left(\nu S^{⁎}+\frac{kU^{⁎}}{\delta }\phi S^{⁎}-\left(1+\frac{kU^{⁎}}{\delta }\right)rE^{⁎}\right)\overset{˜}{g}\left(\frac{S}{S^{⁎}}\right)-\theta E^{⁎}\overset{˜}{g}\left(\frac{EU^{⁎}}{E^{⁎}U}\right)-\nu S^{⁎}\overset{˜}{g}\left(\frac{SU^{⁎}}{S^{⁎}U}\right)-\left(1+\frac{kU^{⁎}}{\delta }\right)\overset{˜}{g}\left(\frac{UVE^{⁎}}{U^{⁎}V^{⁎}E}\right)\\ & -\left(1+\frac{kU^{⁎}}{\delta }\right)rE^{⁎}\overset{˜}{g}\left(\frac{ES^{⁎}}{E^{⁎}S}\right)-\phi S^{⁎}\frac{kU^{⁎}}{\delta }\overset{˜}{g}\left(\frac{SV^{⁎}}{S^{⁎}V}\right)-(\alpha +d)E^{⁎}\frac{kU^{⁎}}{\delta }\overset{˜}{g}\left(\frac{EV^{⁎}}{E^{⁎}V}\right). \end{array}$$

Now, we observe that dL/dt<0 if the following inequalities hold

(26) $$\frac{kU^{⁎}}{\delta }(\theta +\alpha +d+r+\gamma S^{⁎})E^{⁎}\leq dU^{⁎},$$

(27) $$\left(\nu +\phi \frac{kU^{⁎}}{\delta }\right)S^{⁎}\leq \left(1+\frac{kU^{⁎}}{\delta }\right)rE^{⁎}.$$
